# Unlocking high-power aircraft batteries for cryogenic missions via rapid organic base-mediated interfacial kinetics

**DOI:** 10.1093/nsr/nwaf317

**Published:** 2025-08-11

**Authors:** Menglu Li, Hanwen An, Yajie Song, Shengkai Mo, Dakang Peng, Qingsong Liu, Biao Deng, Jiajun Wang

**Affiliations:** MOE Engineering Research Center for Electrochemical Energy Storage and Carbon Neutrality in Cold Regions, School of Chemistry and Chemical Engineering, Harbin Institute of Technology (HIT), Harbin 150001, China; MOE Engineering Research Center for Electrochemical Energy Storage and Carbon Neutrality in Cold Regions, School of Chemistry and Chemical Engineering, Harbin Institute of Technology (HIT), Harbin 150001, China; MOE Engineering Research Center for Electrochemical Energy Storage and Carbon Neutrality in Cold Regions, School of Chemistry and Chemical Engineering, Harbin Institute of Technology (HIT), Harbin 150001, China; MOE Engineering Research Center for Electrochemical Energy Storage and Carbon Neutrality in Cold Regions, School of Chemistry and Chemical Engineering, Harbin Institute of Technology (HIT), Harbin 150001, China; MOE Engineering Research Center for Electrochemical Energy Storage and Carbon Neutrality in Cold Regions, School of Chemistry and Chemical Engineering, Harbin Institute of Technology (HIT), Harbin 150001, China; MOE Engineering Research Center for Electrochemical Energy Storage and Carbon Neutrality in Cold Regions, School of Chemistry and Chemical Engineering, Harbin Institute of Technology (HIT), Harbin 150001, China; Shanghai Institute of Applied Physics, Chinese Academy of Sciences, Shanghai 201204, China; MOE Engineering Research Center for Electrochemical Energy Storage and Carbon Neutrality in Cold Regions, School of Chemistry and Chemical Engineering, Harbin Institute of Technology (HIT), Harbin 150001, China; Chongqing Research Institute of HIT, Chongqing 401135, China

**Keywords:** lithium metal batteries, anion modulation, fast interfacial kinetics, high power density, cryogenic environment

## Abstract

Long-endurance and high-power operation of lithium batteries in cryogenic conditions is important for broader aeronautical applications but is plagued by sluggish and mismatched interfacial kinetics at both electrodes. Herein, we report the concurrent control of the solvation sheath and interfacial chemistry through anion modulation, thereby addressing the challenges associated with charge transfer kinetics. Specifically, lithium bis(trimethylsilyl)amide (LiHMDS) serves as a salt anion adjuvant due to its steric hindrance and electron-donating properties. The spatial effect of LiHMDS induces the construction of weak bidentate coordination structures, promising a fast (de)solvation process. Moreover, electron reconfiguration within the Lewis acid-base (BF_3_-HMDS^−^) promotes the formation of inorganic-rich interphases, eliminating the migration barriers at both electrodes. Consequently, practical pouch cells achieve a high power density of 980.9 W kg^−1^ and an energy density of 310.4 Wh kg^−1^ at −40°C, facilitating high-speed cruising and rapid vertical take-offs and landings of reconnaissance drones in cold environments.

## Teaser

This study presents an anion modulation strategy to address sluggish interfacial kinetics in lithium metal batteries, enabling high power and energy densities to support drone operations in cold environments.

## INTRODUCTION

The low-temperature power performance of high-energy lithium metal batteries (LMBs) is a key enabler of emerging applications such as electric aviation and high-altitude drones [1–3]. However, several challenges remain: (i) slow mass transport resulting in poor ionic conductivity and diffusivity within bulk materials, (ii) increased voltage polarization due to high impedance limitations for Li^+^ (de)solvation and transfer across the electrode-electrolyte interface, and (iii) elevated current density exacerbating the mismatch between ion transportation and interfacial reactions [4]. These issues lead to continuous dendritic deposits and unstable interphases, causing insufficient discharge efficiency at low temperatures [5,6]. Previous research has demonstrated that Li^+^ desolvation and diffusion through the solid electrolyte interphase (SEI) layer are the rate-determining steps [7]. The interfacial charge transfer process is highly dependent on the solvation environment of electrolytes. Thus, the key to accelerating interfacial kinetics lies in regulating the competitive interactions among cations, anions, and solvents.

Strategies including high-concentration electrolytes [8], localized high-concentration electrolytes [9], and weakly solvating electrolytes [10] have been presented to optimize solvation structures. These approaches aim to increase the proportion of anions in the Li^+^ solvation sheath, thereby accelerating the desolvation process and forming an inorganic-rich SEI on the anode. Inorganic species (LiF and Li_3_N) within the SEI can enhance interfacial stability and facilitate Li^+^ transport due to the high modulus and ionic conductivity [11]. Recent advances indicate that rational cathode-electrolyte interphase (CEI) designs can further accelerate Li^+^ desolvation, achieving high energy and power densities under low temperatures [12,13]. Nonetheless, significant voltage drops persist under high current densities or low temperatures, potentially compromising battery usable capacity. Given that charge/discharge kinetics are dictated by the interphases, an optimal electrolyte should create kinetically compatible interphases on anodes and cathodes to enhance capacity utilization at high rates and low temperatures.

Herein, we unlock charge transfer limitations to enable the efficient operation of batteries in cryogenic conditions. An organic base bis(trimethylsilyl)amide (HMDS^−^) with considerable steric hindrance is applied to regulate the anion chemistry in the electrolyte and accelerate interfacial kinetics at both electrodes. The strongly coordinated HMDS^−^ induces the formation of a competitive solvation environment and thus facilitates the (de)solvation process. Additionally, we found a redistribution of electron density in anions after introducing Lewis base HMDS^−^. This behavior refines the tendency of defluorination to result in uniform inorganic-rich interphases that support rapid Li^+^ transport kinetics. The 2.1 Ah Li||LiNi_0.83_Co_0.12_Mn_0.05_O_2_(NCM83) pouch cell with a power density of 980.9 W kg^−1^ delivers 66.7% of the room-temperature capacity at −40°C. Moreover, the assembled batteries exhibit good cycling stability, maintaining performance over 400 cycles at −40°C, indicating the great application potential of such an anion modulation strategy.

## RESULTS AND DISCUSSION

### Anion effect on low-temperature overpotential

**Figure 1.**
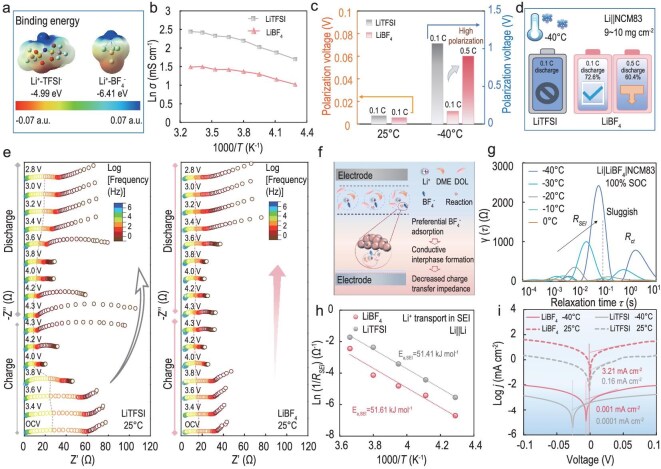
Quantifying charge transfer kinetics and its electrochemical effects. (a) Electrostatic potential (ESP) and binding energy of Li^+^-anion complexes. (b) Temperature dependence of ionic conductivity in LiTFSI and LiBF_4_ electrolytes. (c) The corresponding polarization voltage and (d) capacity retention of Li||NCM83 batteries. (e) *In situ* EIS of Li||NCM83 batteries during the first cycle in different electrolytes. (f) Schematic illustration of LiBF_4_ electrolyte-derived interphases with low interfacial charge-transfer impedance. (g) Temperature-dependent DRT plot derived from EIS data in Li|LiBF_4_|NCM83 batteries. The batteries were activated at room temperature to a fully charged state (100% state of charge, SOC) and subsequently measured across a temperature range from 0°C to −40°C. (h) Corresponding activation energies derived by Arrhenius fitting with different electrolytes for R_SEI_ in Li||Li batteries. (i) Tafel plots of Li plating/stripping in different electrolytes at 25°C and −40°C.

To elucidate the correlation between anions and kinetic polarization at working temperatures, two representative anions of bis(trifluoromethanesulfonyl)imide (TFSI^−^) and tetrafluoroborate (BF_4_^−^) with different dissociation energies were selected for investigation [14]. Based on experimental results, a binary solvent system consisting of 1,3-dioxolane (DOL) and 1,2-dimethoxyethane (DME) in a 7:3 volume ratio was chosen (Figs S1–S3). Electrolytes incorporating LiTFSI and LiBF_4_ salts were named LiTFSI and LiBF_4_ electrolytes. The strong electrostatic attraction between Li⁺ and BF_4_^−^ allows anions to enter the primary solvation shell, reducing Li⁺-solvent coordination. Conversely, the electron-withdrawing capability of the CF_3_SO_2_^−^ group in LiTFSI enhances the delocalization of negative charges on the anionic center, diminishing ion pairing and enhancing salt solubility (Fig. 1a) [15]. As expected, the LiTFSI electrolyte demonstrates a high ionic conductivity (Fig. 1b), yet it suffers from accelerated capacity fade and elevated overpotential at ‒40°C (Fig. S4 and Table S1). In contrast, the Li|LiBF_4_|NCM83 battery maintains 72.6% of its room-temperature capacity with a minimal overpotential of 0.17 V despite exhibiting lower ionic conductivity (Fig. 1c, d).

Given the complex interdependency between overpotential and anion chemistry, *in-situ* electrochemical impedance spectroscopy (EIS) and the distribution of relaxation time (DRT) techniques were employed to investigate the kinetic process of Li||NCM83 batteries during initial cycling [16,17] (Figs 1e and S5). The R_SEI_ of the LiTFSI electrolyte exhibits a notable decrease within the 3.8–4.0 V range, attributed to the high ionic conductivity and the formation of highly conductive sulfur-rich interphases [18]. However, significant variations in R_ct_ are observed in the LiTFSI electrolyte upon charging to 4.3 V (Fig. 1e). During the discharge process, an increase in R_ct_ near 3.6 V induces an elevation in overpotential. The observation is potentially exacerbated under a cryogenic environment [16,19]. The LiBF_4_ electrolytes maintain a consistently lower resistance, indicating rapid interfacial kinetics. From the perspective of anion-specific interfacial behavior, the low R_ct_ of LiBF₄ electrolytes arises from the preferential adsorption of smaller-sized BF_4_^−^ on the cathode surface [20] (Fig. 1f). Such rapid interfacial charge transfer kinetics serve to mitigate electrochemical polarization, thereby enabling a relatively high discharge capacity at −40°C. Notably, when the discharge rate increases to 0.5 C, the LiBF_4_-based battery exhibits a polarization overpotential of 0.90 V and a capacity retention of 60.4% (Fig. 1d).

To clarify the key factors limiting the usable capacity at high-rate conditions, we conducted the temperature-dependent DRT to analyze the impedance evolution in the Li||NCM83 batteries [17,21]. Compared with LiTFSI, the R_ct_ of the LiBF_4_ electrolyte is smaller, particularly at lower temperatures (Figs 1g and S6). The resistance associated with Li^+^ transport through the SEI dramatically increases at extremely low temperatures in both electrolytes. It should be noted that the typical time constant (τ) for specific kinetic processes represents a distribution range rather than an accurate value. As temperature decreases, each electrochemical step demonstrates progressively sluggish kinetics, leading to increased interfacial impedance and a corresponding shift of τ toward higher values [22,23].

Additionally, temperature-dependent EIS was performed on Li||Li batteries to effectively eliminate interference from the cathode. The Arrhenius fitting of R_SEI_ reveals high activation energies (E_a_) in both electrolytes, indicative of sluggish kinetics at the anode (Figs 1h and S7). Additionally, the exchange current density (*j*) in the LiBF_4_-based anode exhibits a substantial decrease from 3.21 mA cm^−2^ to 0.001 mA cm^−2^ with decreasing temperature, further highlighting slow Li^+^ transfer kinetics across the electrolyte/anode interface (Fig. 1i). Although the LiBF_4_-based battery shows accelerated interfacial kinetics at the cathode, the kinetically matched interphases on both electrodes are adversely lacking (Fig. S8).

### Optimizing the anion-rich solvation environment

The involvement of anions in the solvation sheath plays a fundamental role in governing the physicochemical functions of electrolytes. Although LiBF_4_ has been proven effective in diminishing the interfacial kinetic barrier at the cathode, its insufficient passivation capability toward the anode remains a critical limitation [24]. Due to the thermodynamic tendency of LiBF_4_ to decompose into LiF and the strong Lewis acid BF_3_, the latter undergoes complexation with trace water to generate protic acid, thereby inducing electrolyte decomposition and inhibiting the formation of a stable interphase. In principle, the introduction of a suitable Lewis base can modulate the decomposition behavior of LiBF_4_, enabling the formation of kinetically matched cathode and anode interphases while preserving the low-temperature adaptability of DOL. Lithium bis(trimethylsilyl)amide (LiHMDS), a strong organic base with a low redox potential (Fig. S9), has been demonstrated to facilitate the formation of an (electro)chemically robust CEI for high-temperature operation [25]. However, its influence on interfacial kinetics at low temperatures remains inadequately explored.

**Figure 2.**
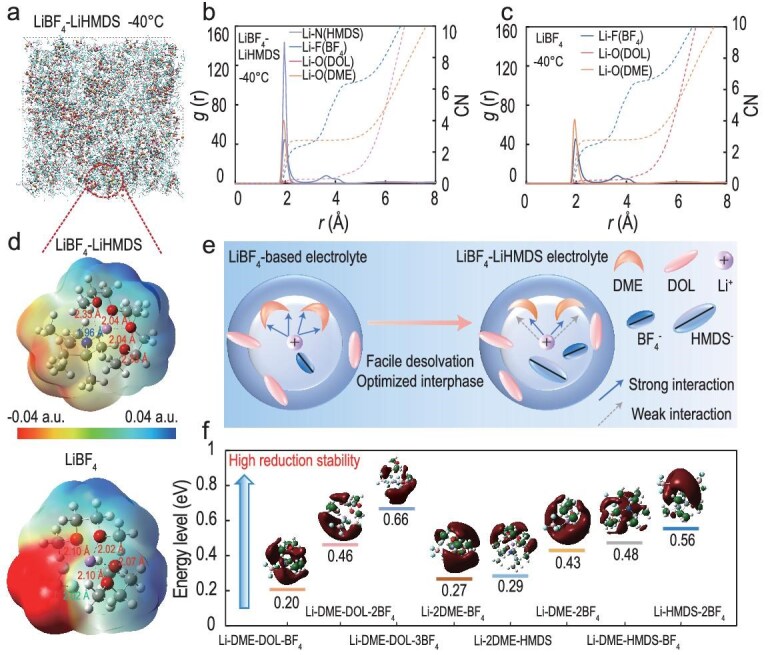
Insights into electrolyte solvation structures and properties. (a) Snapshots of distributions of anions and solvents in the LiBF_4_-LiHMDS electrolyte. RDF of different components at −40°C in (b) LiBF_4_-LiHMDS and (c) LiBF_4_ electrolyte. (d) The corresponding main solvation structure from MD simulations and ESP calculations of different electrolytes. (e) Schematic illustration of solvation structures and effects derived from MD analysis in different electrolytes. (f) LUMO energy of representative solvation configurations.

Therefore, LiHMDS was introduced as an anion modulator into the LiBF_4_ electrolyte, resulting in the preparation of the LiBF_4_-LiHMDS electrolyte. This modification maintains a sufficient bulk ionic conductivity of 2.49 mS cm^−1^ for effective ion transport at −40°C (Fig. S10). Molecular dynamics (MD) simulations were performed at 25°C and −40°C to understand the solvation configuration. Snapshots of solvation boxes for two different electrolytes are displayed in Figs 2a and S11. From the radial distribution functions (RDFs) (Figs 2b, c, and S12), the distance of Li-N (HMDS) peak in the LiBF_4_-LiHMDS electrolyte is 1.96 Å. The coordination numbers of Li-F(BF_4_) decrease to 2.57, implying that the HMDS^−^ is involved in the first solvation sheath and partially substitutes BF_4_^−^ [26]. A similar trend can be observed in the peaks of BF_4_^−^ located at 761 cm^−1^ in Raman spectroscopy (Fig. S13) [27]. The extracted typical solvation structures further complement the results of competitive interactions, and their first solvation structures are all anion-rich (Fig. S14).

The bond lengths of different typical solvation structures were calculated. The strong bidentate coordination structure is disrupted once HMDS^−^ participates in the coordination with Li^+^, accompanied by different Li^+^-O (DME) bond lengths of 2.33 Å and 2.04 Å, respectively (Fig. 2d). Notably, the 2.33 Å value is much longer than the bond length observed in the Li-2DME-BF_4_ (2.10 Å). The coordination mode was also verified by Raman spectroscopy of the electrolytes (Fig. S15) [28]. Such an asymmetric coordination configuration can successfully maximize the steric spatial effect of molecules, endowing a more facile desolvation process without compromising ion migration over a wide temperature range (Fig. 2e).

ESP calculations in Fig. 2d show a blue shift in LiBF_4_-2DME structure, indicating that more positive charges are distributed at the outer end of DME molecules. This shift enhances electron acceptance and preferential reduction [29], forming a crystalline interphase with high interfacial resistance. Conversely, the LiBF₄-LiHMDS electrolyte suppresses unwanted solvent reduction, favoring anion-derived SEI formation. The calculated lowest unoccupied molecular orbital (LUMO) energy levels further complement the results on the reduction stability with the incorporation of LiHMDS (Fig. 2f). The reduction behavior of the electrolytes was investigated using linear sweep voltammetry (LSV) on Li||Cu batteries at 25°C and −40°C (Fig. S16). The LiBF₄-LiHMDS electrolyte exhibits a lower onset reduction potential of 1.40 V compared to 1.45 V for the LiBF_4_ electrolyte, along with weaker signals associated with solvent decomposition. Similar trends were observed at −40°C. These observations are attributed to the formation of a robust SEI in the LiBF₄-LiHMDS system, which effectively mitigates the decomposition of solvents.

### Kinetics evolution and formation mechanism of SEI

**Figure 3.**
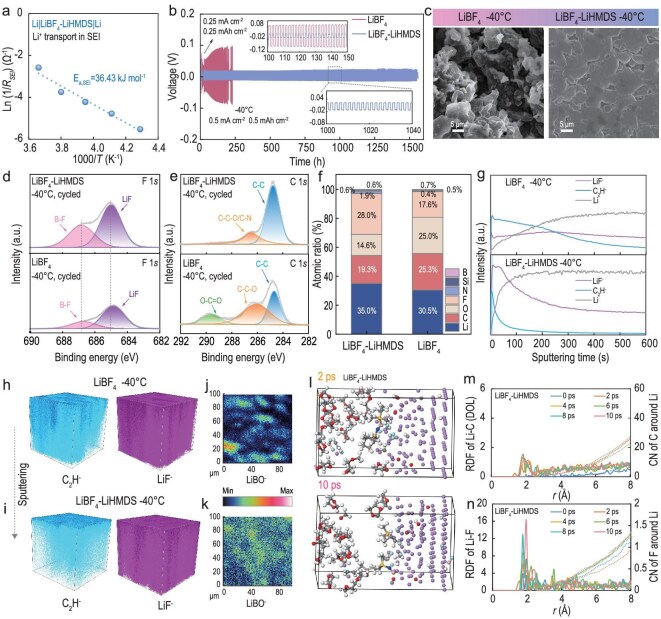
Effect of LiHMDS on Li anode at −40°C. (a) Corresponding activation energies for R_SEI_. (b) Cycling stability of Li||Li employing LiBF_4_ and LiBF_4_-LiHMDS electrolytes. The batteries were initially cycled at 0.25 mA cm^−^² (0.25 mAh cm^−^²), followed by long-term cycling at an increased current density of 0.5 mA cm^−^² (0.5 mAh cm^−^²). (c) SEM images of cycled Li metal in different electrolytes. XPS analysis of (d) F 1*s*, (e) C 1*s* and (f) corresponding atomic concentration contents of different electrolytes. The interphases were formed at room temperature and cycled at 0.1 C under −40°C. (g) Depth profiles of various species of secondary ion fragments obtained by sputtering and TOF-SIMS analysis of the SEIs formed in different electrolytes. TOF-SIMS three-dimensional images of the C_2_H^−^ and LiF^−^ species in the SEIs with the (h) LiBF_4_ and (i) LiBF_4_-LiHMDS electrolytes. The corresponding two-dimensional distributions of LiBO^−^ in the (j) LiBF_4_ and (k) LiBF_4_-LiHMDS electrolytes. The interphases were formed at room temperature and cycled at 0.1 C under −40°C. (l) The snapshots of 2 ps and 10 ps AIMD simulation of the adsorption and decomposition of LiBF_4_-LiHMDS solvation complex on the Li surface. (m) Li-C (DOL) RDF evolution during the simulation process. (n) Li-F RDF evolution during the simulation process.

To clarify the effects of LiHMDS on electrochemical kinetics, temperature-dependent EIS was measured in Li||Li symmetric batteries. The activation energy for Li^+^ transport through the SEI (E_a, SEI_) in the LiBF_4_-LiHMDS electrolyte is reduced from 51.61 to 36.43 kJ mol^−1^ when compared to the LiBF_4_ electrolyte (Figs 1h, 3a, and S17). Such enhanced interfacial kinetics are advantageous for suppressing the incomplete reaction of the solvents and salts under subzero conditions [30]. This result is simultaneously corroborated by the improved exchange current density (Fig. S18). The Li^+^ transference number for the LiBF_4_-LiHMDS electrolyte is 0.58, whereas that for the LiBF_4_-based electrolyte is only 0.41 (Fig. S19). This elevated transference number signifies a more efficient transport mechanism for Li⁺ within the LiBF_4_-LiHMDS electrolyte, thereby substantially diminishing electrode polarization [31].

The compatibility of the Li anode with different electrolytes was evaluated at −40°C by galvanostatic plating/stripping profiles in Li||Li symmetric batteries (Fig. 3b). The uneven distribution of SEI components and morphology accelerates dendrite formation at low temperatures (Fig. 3c). Comparatively, the LiBF_4_-LiHMDS electrolyte achieves a more stable and lower overpotential up to 1560 hours. The dense and uniform deposition layer derived from LiBF_4_-LiHMDS helps accelerate Li^+^ transport, which favors the uniform Li^+^ flux and results in even Li metal deposition. Moreover, the assembled Li|LiBF_4_-LiHMDS|Li battery always shows less overpotential over a wide range of current densities from 0.25 to 3 mA cm^−2^ at −40°C (Fig. S20). The Aurbach coulombic efficiency (CE) tests [32] further demonstrate the good reversibility in Li deposition/stripping using LiBF_4_-LiHMDS, showing a substantially improved CE (97.38%) compared with that using LiBF_4_ (94.43%) and LiTFSI (74.76%) at −40°C. Furthermore, it was found that the LiBF_4_-LiHMDS electrolyte exhibits smooth Li deposition/stripping curves and small overpotential, which was not shared by the LiBF_4_ and LiTFSI systems, indicative of soft shorting events (Fig. S21).

X-ray photoelectron spectroscopy (XPS) was conducted to investigate the chemical compositions of the SEI formed at low temperatures. The inorganic components (Li_x_BF_y_ and LiF signals in the F 1*s* spectrum) [27] are enriched in the LiBF_4_-LiHMDS electrolyte-derived SEI (Fig. 3d), which can significantly enhance the ionic conductivity of the interphase. The Si/Si-C, C-N species and excellent Li^+^ conductive components (such as Li_3_N) are regarded as the reductive decomposition products of LiHMDS [25,33], suggesting that LiHMDS can participate in SEI formation and exhibit a long-lasting protective effect on interfacial stability even after extended cycling (Figs S22 and S23). By contrast, the higher presence of organic components (C-C-O 286.5 eV, and O-C=O 289.7 eV) [34] in C 1*s* spectra was detected on the LiBF_4_ electrolyte-derived SEI (Fig. 3e). The results suggest that the SEI formed with LiBF_4_ is insufficient in protecting the Li metal anodes, resulting in the solvents’ continuous decomposition. The elemental content of different SEIs was compared via quantitative XPS analysis (Fig. 3f). In short, the inorganic-rich and organic-deficient features make the LiBF_4_-LiHMDS–derived SEI robust and conductive during cycling, thereby accelerating the transport of Li^+^ and mitigating the persistent degradation of electrolytes.

Time-of-flight-secondary ion mass spectrometry (TOF-SIMS) was further performed to visualize the chemical evolution process at the anode interface. The depth-profiled data show that organic moieties (C_2_H^−^) mainly concentrate on the LiBF_4_ electrolyte-derived SEI (Figs 3g, h, and S24). Notably, the rapid decay of the C_2_H^−^ peak signals can be detected in the LiBF_4_-LiHMDS electrolyte as sputtering prolongs, which implies the organic component only in the outermost layer of the SEI (Fig. 3i). In addition, the two-dimensional overlay images reveal an inhomogeneous spatial distribution of LiBO^−^ fragments in the LiBF_4_ electrolyte-derived SEI (Fig. 3j), which is likely due to the sluggish kinetics of the BF_4_^−^ decomposition at low temperatures [35]. By contrast, the homogenous LiBO^−^ and LiBF^−^ signal mapping formed by the LiBF_4_-LiHMDS electrolyte indicates that the anion-mediator LiHMDS governs the interfacial behaviors and efficiently passivates the electrochemical reaction sites (Figs 3k and S25). The peak of Li_3_N^−^ (*m/z* = 35) was identified in the ion mass spectrometry data (Fig. S26), aligning well with the XPS data. The grain boundaries among multiple inorganics (e.g. LiF, Li_3_N, Li_x_BF_y_, and LiBO_x_) significantly enhance the concentration of ionic carriers and establish efficient channels for Li^+^ transport [36,37].

It has been demonstrated that LiBF_4_ can decompose into LiF and the Lewis acid BF_3_, with the latter readily coordinating with H_2_O to generate the protic acid H⁺(BF_3_OH)⁻ [38]. The protons initiate the ring-opening of DOL by attacking its oxygen lone pairs, leading to carbocation formation and subsequent polymerization (Fig. S27) [39]. Notably, the initial SEI formed by LiBF_4_ is not as effective at passivating the electrode due to the hydrolysis of LiBF_4_ [24]. Therefore, it is important to consider specific surface conditions and potential impurities when examining the interfacial reactions between the anode and electrolyte. To understand the role of LiHMDS in SEI formation, the binding energy and bond length of different complexes were studied using DFT calculations. The BF_3_ presents the highest binding energy with HMDS^−^ (Fig. S28), implying that the formation of H^+^(BF_3_OH)^−^ is inhibited more effectively owing to the electron-donating nature of the HMDS^−^ functional group. Additionally, the binding distance between the N (HMDS) site and B (BF_3_) (1.58 Å) is shorter than that of H_2_O (H-F 2.59 Å), accompanied by the longer bond length of B-F (1.41 Å) in BF_3_-HMDS^−^. The B-F bonding electrons in Lewis acid (BF_3_) tend to shift towards the F side after combining with the Lewis base (LiHMDS), contributing to an enhanced affinity for Li^+^ [35].

The Ab Initio Molecular Dynamics (AIMD) simulation trajectory suggests that the B-F(BF_3_-HMDS^−^) bond is further weakened by the electrostatic attraction of Li^+^, and the electron-rich F atom is then captured to form LiF (Figs 3l and S29) [40,41]. In the simulation employing the LiBF_4_-LiHMDS electrolyte, the increasing and closer Li-F peaks along the simulation timescales indicate that the exterior Li atoms are bonded with more F atoms and fewer C atoms (Figs 3m, n, and S30). Formation of the organic-rich SEI accompanies the sustained decomposition of solvent molecules in LiBF_4_-based electrolytes, thus hindering the continuity of protective inorganic layers. Significant variations in SEI components occur during the trajectory, suggesting that the SEI formed is unstable (Figs S31 and S32). The results are consistent with the XPS and TOF-SIMS analysis. In brief, the undesirable decomposition of solvents is fundamentally suppressed based on intermolecular electron reconfiguration, resulting in a uniform and dense SEI dominated by LiF on Li metal anodes.

### Electrochemical kinetics analysis and characterization of cathodes

**Figure 4.**
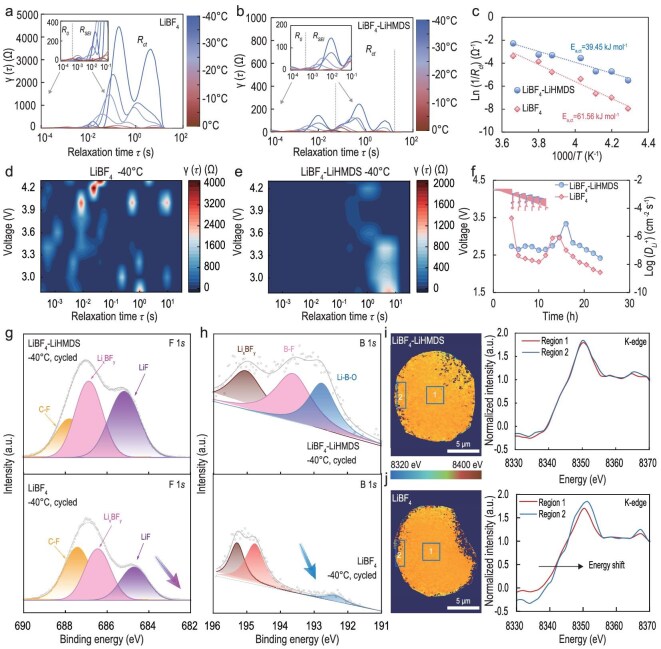
Characterization of NCM83 cathodes. Temperature-dependent DRT plots derived from EIS data in Li||NCM83 batteries for (a) LiBF_4_ and (b) LiBF_4_-LiHMDS electrolytes. The batteries were cycled at −40°C to a fully charged state, followed by electrochemical characterization at different temperatures. (c) Corresponding activation energies derived by Arrhenius fitting for R_ct_ with the LiBF_4_ and LiBF_4_-LiHMDS electrolytes. *In-situ* DRT data representing the second discharge of Li||NCM83 batteries at −40°C with the (d) LiBF_4_ and (e) LiBF_4_-LiHMDS electrolytes. (f) Voltage curves and the diffusion coefficient of Li^+^ from the GITT measurement on the batteries with LiBF_4_ and LiBF_4_-LiHMDS electrolytes at −40°C after two formation cycles. XPS spectra of (g) F 1*s* and (h) B 1*s* of NCM83 cathode surfaces in different electrolytes. The interphases were formed at room temperature and cycled at 0.1 C under −40°C. The battery was disassembled at a fully discharged state. TXM mapping and the average XAS spectra of the whole particle after cycling at −40°C in (i) LiBF_4_-LiHMDS and (j) LiBF_4_ electrolytes.

The kinetic process of Li||NCM83 batteries during initial room-temperature cycling was also investigated via *in situ* EIS (Fig. S33). The results demonstrate that the LiBF_4_-LiHMDS system can construct a low-impedance and stable electrode-electrolyte interface, thereby providing critical assurance for the stability of the battery. The temperature-dependent DRT profiles reveal that the R_ct_ of the LiBF_4_-based battery is an order of magnitude higher than that of the LiBF_4_-LiHMDS battery, underscoring the significant role of the anion modulator in facilitating the charge transfer process at low temperatures (Figs 4a, b, and S34). The enhanced charge transfer kinetics in the LiBF_4_-LiHMDS battery are further substantiated by the significantly lower activation energy required for this process, measured at 39.45 kJ mol^−1^, in stark contrast to the higher value of 61.56 kJ mol^−1^ calculated for the LiBF_4_ battery (Fig. 4c). To visualize the trends of R_ct_ and R_SEI_ during discharge at −40°C, we conducted *in situ* EIS-combined DRT mapping on the Li||NCM83 battery (Fig. 4d, e). In contrast to the LiBF_4_ battery, the smaller R_SEI_ and R_ct_ observed in the LiBF_4_-LiHMDS battery suggest a more facile Li^+^ migration through the SEI and an enhanced Li^+^ (de)solvation process. Galvanostatic intermittent titration technique (GITT) measurements reveal that the Li^+^ diffusion coefficient (D_Li^+^_) of the LiBF_4_-LiHMDS cathode is slightly higher than that of the LiBF_4_ electrolyte (Fig. 4f). This finding further underscores the importance of interfacial kinetics over bulk transport properties in optimizing low-temperature performance.

Precise control of anion chemistry is crucial for designing an effective CEI, ensuring optimal battery performance and stability across the entire operating potential and temperature range. The DFT calculations show that the highest occupied molecular orbital (HOMO) level of the LiBF_4_-LiHMDS is higher than that of other components of the electrolyte (Fig. S35). Furthermore, the HOMO is mainly located on the HMDS^−^ instead of solvents in the LiBF_4_-LiHMDS electrolyte. As shown in Fig. 4g, h, highly robust and conductive components mainly from anion decomposition were detected in the cycled LiBF_4_-LiHMDS cathode at −40°C, such as LiF (685 eV), Li_x_BF_y_ (193.8 eV), and Li-B-O (192.7 eV). In the Si 2*p* spectrum, the peaks of Si-C (101.8 eV) [25] only appear on the LiBF_4_-LiHMDS cathode (Fig. S36). Meanwhile, the signals C-C-O (286.5 eV) and C=O (287.8 eV) [34] are weakened, verifying that the introduction of LiHMDS significantly suppresses continuous solvent decomposition on the cathode surface (Fig. S37). TOF-SIMS characterization was performed on cycled cathodes to further identify CEI composition. Species containing CN⁻ and Li_3_N⁻ are detected at *m/z* = 26 and *m/z* = 35, respectively (Fig. S38). Furthermore, the LiHMDS preferentially decomposes on the cathode to improve the CEI while modulating the anion behavior and improving the SEI at the anode as demonstrated by inductively coupled plasma-optical emission spectrometry (ICP-OES), resulting in a well-balanced comprehensive performance (Table S2).

Note that the low-temperature operation of batteries can result in irreversible structural damage in active electrodes, negatively impacting subsequent battery cycling performance [42]. As observed in Fig. S39, prominent cracks were evident in LiBF_4_-based NCM83 particles after cycling, resulting in extensive electrolyte penetration and particle pulverization [43]. In contrast, no discernible cracking was detected in NCM83 particles cycled within the LiBF_4_-LiHMDS electrolyte at low temperature. Additionally, synchrotron transmission X-ray microscopy (TXM) was employed to visualize the spatial morphological and chemical-structural changes in sample particles post-cycling at −40°C [44]. The X-ray absorption spectrum (XAS) absorption edge of the interfacial region in LiBF_4_-based particles exhibits a positive shift relative to the core region, indicative of a heterogeneous state of charge (SOC) distribution across spatial dimensions following low-temperature charging. Such reaction heterogeneity and high irreversibility are detrimental to the low-temperature performance of the battery due to increased interfacial resistance and incrementally sluggish interfacial kinetics (Fig. 4i, j) [45]. Conversely, the uniform chemical phase distribution observed in the LiBF_4_-LiHMDS system is primarily attributed to the enhanced formation of a monolithic and inorganic-rich (B-O/Si-C) CEI.

### Electrochemical performance under low temperatures

**Figure 5.**
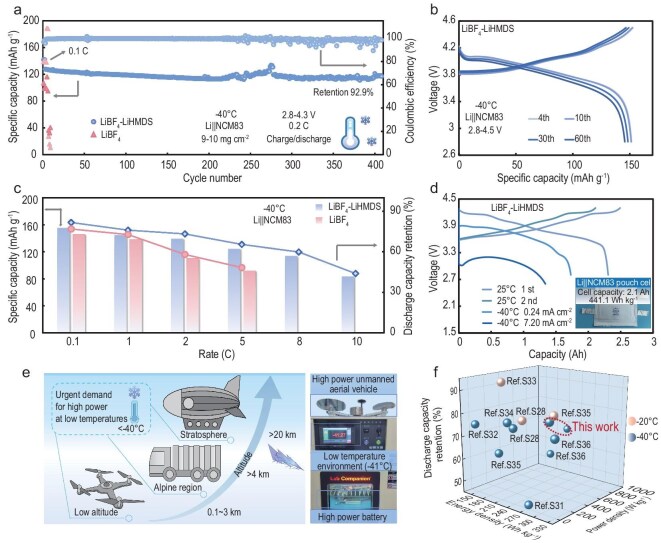
Electrochemical performances of Li||NCM83 batteries using different electrolytes at −40°C. (a) Cycling behavior of Li||NCM83 batteries with different electrolytes. (b) The voltage profiles of the Li|LiBF_4_-LiHMDS|NCM83 battery at a voltage of 4.5 V. (c) The discharge capacity and retention at different discharging rates. (d) Charge/discharge profiles of Li||NCM83 pouch cells under practical conditions (areal capacity: 2.4 mAh cm^−2^, lean electrolyte 1.6 g Ah^−1^). Inset is the optical image of the 2.1 Ah pouch cell. (e) Optical images of an electric drone powered by the Li||NCM83 pouch cell using LiBF_4_-LiHMDS electrolyte and emerging applications. (f) Comparison of pouch cell-level (output) power density, discharge capacity retention, and energy density at low temperatures between recently reported work and the present work. ‘Ref. Sx’ represents Supplementary reference x.

To highlight the role of LiHMDS in accelerating the charge transfer process, we assembled Li||NCM83 batteries and tested them in harsh conditions. As shown in Fig. 5a, the Li|LiBF_4_-LiHMDS|NCM83 battery exhibits high capacity retention of 92.9% after 410 cycles at 0.2 C under −40°C. The cycle-related EIS demonstrates the effectiveness of continuous protection and fast interfacial kinetics enabled by the inorganic-rich interphases (Fig. S40). In contrast, LiBF_4_-based batteries quickly failed after only a few cycles. Practical application also requires that the electrolyte work with cathode materials, especially high-voltage oxides. LSV measurement using Li||Al reveals distinct electrochemical stability windows for the investigated electrolytes at 25°C and −40°C (Fig. S41). The oxidation decomposition threshold voltage of the LiBF₄-LiHMDS electrolyte further increased to 4.9 V at −40°C, which is significantly higher than that of the LiBF_4_ electrolyte under the same conditions. As such, the long-term stability of Li||NCM83 with the LiBF_4_-LiHMDS electrolyte still outperformed at a higher voltage of 4.5 V (Figs 5b and S42). In addition to good low-temperature performance, LiBF_4_-LiHMDS batteries also show stable cycling performance at room temperature (Fig. S43).

The enhanced interfacial kinetics observed at both electrodes prompt a comprehensive evaluation of the fast-discharging performance of the LiBF_4_-LiHMDS electrolytes. Our investigation reveals that the Li|LiBF_4_-LiHMDS|NCM83 battery maintains 88.4% of its initial capacity at a high discharge rate of 20 C at 25°C (Fig. S44). Furthermore, we conducted comparative tests on Li||NCM83 batteries utilizing various electrolytes under cryogenic environments. The results indicate that both electrolytes exhibit high-capacity retention at 0.1 C under −40°C. However, the LiBF_4_-based battery displays a higher overpotential above 2 C owing to the loss of Li and consumption of electrolytes (Fig. S45). More strikingly, the Li|LiBF_4_-LiHMDS|NCM83 batteries achieve a discharge capacity of 84 mAh g^−1^ even at an elevated rate of 10 C [9] (Fig. 5c). This is ascribed to the similar kinetics between the NCM83 cathode and Li metal anode over a wide temperature range, thereby encouraging the fast discharging of LMBs.

To demonstrate the practical efficacy of anion modulator LiHMDS at low temperatures, the pouch cells comprising high-loading NCM83 with lean electrolytes were tested. At room temperature, the Li|LiBF_4_-LiHMDS|NCM83 pouch cell achieves a specific energy density of 441.1 Wh kg^−1^ at 0.1 C and displays cycling stability by maintaining 92.7% capacity after 92 cycles (Fig. S46). Even at −40°C, the cell still provides a high energy density of 310.4 Wh kg^−1^. The Li|LiBF_4_-LiHMDS|NCM83 pouch cell retained 95.4% of its capacity after 18 cycles at 0.1 C under −30°C (Fig. S47). Furthermore, the pouch cell can retain 66.7% of its room-temperature capacity when discharged at 7.20 mA cm^−2^ under −40°C (Fig. 5d). This high output current density enables the pouch cell to achieve a high-power density of 980.9 W kg^−1^ with an energy density of 217.9 Wh kg^−1^ (Fig. S48). This outstanding performance indicates that the system maintains significant advantages in sustaining high-rate capabilities while preserving elevated levels of usable energy density. Moreover, the LiBF_4_-LiHMDS electrolyte allows the pouch cell to power an electric drone at −40°C after room temperature charging. Compared to previously reported low-temperature lithium batteries, the remarkable usable capacity and power density highlight the potential of the LiBF_4_-LiHMDS electrolyte for applications in emerging scenarios (Fig. 5e, f). The enhanced electrochemical performance of LiBF_4_-LiHMDS substantiates the effectiveness of our strategy in modulating anion chemistry.

## CONCLUSION

In summary, we have elucidated the correlation between interfacial kinetics and low-temperature performance modulated by various anion chemistries. As a proof of concept, we demonstrate that employing LiHMDS as an anion regulator simultaneously addresses challenges associated with Li⁺ (de)solvation and interfacial migration. The unique coordination mode induced by the bulky size of LiHMDS enables a rapid desolvation process while preserving the high dielectric constant of solvents. Moreover, the electron transfer within Lewis acid-base complexes promotes the cleavage of B-F bonds, thereby facilitating the formation of fluoride-enriched interphases that enable rapid Li^+^ migration. This work elucidates the correlation between the anion chemistry and the redox behavior of electrolytes, interfacial kinetics, and anodic/cathodic protection. These advancements enable practical pouch cells to sustain continuous high-rate discharge at −40°C, ensuring rapid start-up and high-power operation of specialized equipment in cryogenic environments.

## METHODS

All the chemicals used in this work were purchased from commercial vendors. Lithium bis(trifluoromethanesulfonyl)imide (LiTFSI), lithium tetrafluoroborate (LiBF_4_), 1,3-dioxolane (DOL), 1,2-dimethoxyethane (DME), and lithium hexamethyldisilazide (LiHMDS) were purchased from Sigma-Aldrich. The solvents were dried with activated molecular sieves for at least 24 hours before use. First, DOL and DME were well-mixed according to stoichiometry. Then, LiHMDS was introduced into the mixture at a concentration of 2.5% relative to the total electrolyte mass, followed by stirring at ambient temperature for 2 hours. Finally, LiBF_4_ was dissolved entirely by stirring to obtain the LiBF_4_-LiHMDS electrolyte (2 M LiBF_4_ in DOL:DME 7:3 v/v). The preparation process of other electrolytes was similar to the aforementioned method, except for the exclusion of LiHMDS. The electrolyte preparation was performed in an argon-filled glovebox with H_2_O and O_2_ levels <0.01 ppm. Detailed materials and methods are available in the online Supplementary Data.

## Supplementary Material

nwaf317_Supplementary_data
