# Supplementary material for: Unlocking high-power aircraft batteries for cryogenic missions via rapid organic base-mediated interfacial kinetics
Source: Natl Sci Rev. 2025 Aug 11;12(9):nwaf317. doi: 10.1093/nsr/nwaf317 (PMC12421576; doi:10.1093/nsr/nwaf317)
Supplement: nwaf317_Supplementary_data [file nwaf317_supplementary_data.pdf]

SUPPLEMENTARY INFORMATION

Unlocking high-power aircraft batteries for cryogenic missions via rapid organic base-mediated interfacial kinetics

Menglu Li, Hanwen An, Yajie Song, Shengkai Mo, Dakang Peng, Qingsong Liu, Biao Deng, Jiajun Wang*

METHODS

Electrode preparation. $LiNi_{0.83}Co_{0.12}Mn_{0.05}O_2$ (NCM83) cathodes PVDF and Super p were added to N-Methyl pyrrolidone (NMP, Aladdin, 98%) solvent in a mass ratio of 94:3:3, and the uniform electrode slurry was obtained through a planetary agitator. Then the obtained slurry was coated on the aluminum current collector. Subsequently, the electrodes were initially dried at 80℃ for 4 h and subsequently dried at 110 ℃ for 12 h under vacuum to eliminate residual solvent. Finally, to prepare a dense composite cathode, press the dried cathode to 1 MPa cm^{-2}. The 1 C rate for NCM83 cathodes is defined as 200 mA g^{-1}, calculated based on its nominal capacity of 200 mAh g^{-1}.

Battery assembly. The CR2025 Li||NCM83 coin batteries were assembled in an Ar-filled glove box (O_2 and H_2O < 0.01 ppm) using 50 μL $LiBF_4$-based electrolyte with/without LiHMDS. The thickness of lithium foil is 450 μm and the diameter is 15.6 mm. The diameter of the cathode is 12 mm and the thickness of the separator is 38 μm. The high loading of active material is 8.0~10.0 mg cm^{-2}, while another loading level is 3.0~4.0 mg cm^{-2}.

For the assembly of pouch cells, the cathode features a double-sided coating with a loading capacity of 24.0 mg cm^{-2} (12.0 mg cm^{-2} per side), the anode is made of metallic lithium (thickness: 40 μm), and the separator consists of a (16+2+2) μm double-sided ceramic (Al_2O_3) coated PP film. The single cathode sheet measures 122×55 mm^2, while the anode spans 126×57.5 mm^2. A 2.1 Ah pouch cell, comprising 7 cathodes and 8 anodes, is prepared using a Z-shaped lamination process. The cell is finally encapsulated with aluminum-plastic film, with a liquid injection volume of 1.6 g Ah^{-1}. The final battery weight is 20.38 g, and its discharge energy (at 0.1 C) is 8.99 Wh, resulting in an energy density of 441.1 Wh kg^{-1}.

Electrochemical measurements. Galvanostatic charge/discharge measurements were performed using the Neware battery test system (BTS-2004, Shenzhen, China) under different temperatures and voltage ranges of batteries. For the low-temperature experiments, Li||NCM83 batteries were first activated for one cycle at room temperature and then placed in a freezer (Lab companion PG-80) at the targeted temperature. Pouch cells were tested under 1 MPa pressed by the splint. The ionic conductivity of electrolytes was estimated by conductivity meter (DDSJ-308F).

The t_{Li+} was conducted by direct-current (DC) polarization of the Li symmetric battery with the DC voltage of 10 mV at 25 ℃. And t_{Li+} was calculated by equation (1):

$$t_{Li^+} = \frac{I_s(\Delta V - I_0 R_0)}{I_0(\Delta V - I_s R_s)} \quad (1)$$

where ΔV is the applied voltage (10 mV), I_0 and I_s are the initial and steady current through the Li||Li batteries, respectively, R_0 and R_s are the initial and steady resistances of the Li||Li batteries obtained by AC impendence, respectively.

The electrochemical window of the different electrolytes was determined via linear sweep voltammetry (LSV) technique using a Li || Al half-cell, scanned from OCV to 6.0 V at 0.5 mV s^{-1}. The Li || Cu half-cell was also tested from OCV to 0 V at a scan rate of 1.0 mV s^{-1} for reductive stability. Tafel plot was obtained from linear sweep voltammetry measurement using Li||Li symmetrical batteries at 0.5 mV s^{-1}. The values of exchange current density were calculated using the Tafel equation: $\eta = a + b\log(I)$, where η and I are the potential and current, respectively, and a and b are the constant that could be acquired after fitting the data.

For Li metal average CE determinations, the standard Aurbach protocol was conducted on the Li||Cu half battery [1]. We adopted the following standard protocol:1) Initial formation cycles was conducted by plating 4 mAh cm^{-2} Li on Cu foil at 0.5 mA cm^{-2}, then stripping to 1.0 V; 2) Initial Li source were plating again of 4 mAh cm^{-2} on Cu at 0.5 mA cm^{-2}; 3) Perform 10 cycles at 1 mAh cm^{-2} and 0.5 mA cm^{-2}; (4) Finally stripping residual Li from Cu to 1.0 V. The CE was calculated as the ratio of total stripped to plated capacity.

In GITT measurements, the batteries were cycled at a rate of 0.1 C with 30 min pulse time and 1 h rest time. EIS test was applied after cycling with an amplitude of 5 mV in the frequency range from 0.01 Hz to 1000 kHz.

Characterization and Measurements. Raman spectra were recorded on a Renishaw Raman spectrometer. The morphology of the cathodes and powders was observed by field-emission scanning electron microscopy (SEM, Phenom Pro), and the smooth cross-section of the cathode was achieved by a cross-section ion polisher (accelerating voltage: 2~6 kV, beam spot size: 500 μm). The chemical composition was analyzed

by XPS (ESCALAB 250Xi). The corresponding atomic concentration contents were mapped using ChiPlot (https://www.chiplot.online/gene_cluster.html). To further investigate the composite and microstructure of SEI, time-of-flight secondary ion mass spectrometry (TOF-SIMS, IONTOF GmbH, Münster, Germany) was employed. The 3D data was obtained on the area of 100 μm × 100 μm. The measurements were conducted in negative modes. Inductively coupled plasma-optical emission spectrometry (ICP-OES) was performed on the Agilent 5110 to measure the consumption of the electrolyte additive at both anode and cathode interfaces.

Transmission X-ray Microscopy (TXM) and data analysis. The synchrotron X-ray-based TXM was recorded at Shanghai Synchrotron Radiation Facility (SSRF), beamline BL18B. At above 15 keV, for ~180 projections over an angular range of 180° with a field of view of 20 × 20 μm^2. The raw data obtained is tomographically aligned and reconstructed [2]. The 3D visualization and data analysis were performed using the commercial software package Avizo (Thermo Fisher Scientific, Waltham, Massachusetts, USA) [3].

Computational Details

cMD simulations. GROMACS 2023.02 software package was used for molecular dynamics simulation, and all molecules were described by OPLSAA force field [4]. The simulation models were built in accord with the experimental composition, that is, $LiBF_4$-based electrolyte (200 $LiBF_4$, 453 DME molecules, 1255 DOL molecules) $LiBF_4$-LiHMDS electrolyte (200 $LiBF_4$, 453 DME molecules, 1255 DOL molecules, and 5 LiHMDS) randomly placed in a periodic box with a volume of 125 nm^3. The initial configurations of all the simulated systems were obtained by mixing the components uniformly with Packmol software package. First, the steepest gradient method is used to minimize the energy of 3000 steps to eliminate the unreasonable overlap between atoms. Then 100 ps relaxation was performed at 298.15 K under NVT and NPT systems with an integral time step of 1.0 fs respectively. Finally, 20 ns production simulation was performed under NPT system. In MD simulation, periodic boundary conditions are used in all three directions, and the time step is 2.0 fs. The temperature is maintained at 298.15 K or 233.15 K with a V-rescale thermostat with a coupling time of 0.5 ps, and the pressure is controlled at 1 bar with a Parrinello-Rahman constant voltage regulator with a coupling time of 2.0 ps. All

hydrogen-containing chemical bonds (C-H, O-H) were constrained by LINCS algorithm. The particle grid Ewald (PME) method was used to deal with the long-distance electrostatic interactions, and the short-range electrostatic and vdW interactions were calculated using a cut-off value of 1.2 nm. GROMACS software package was used for tracking post-processing.

Quantum chemistry calculations. The DFT implanted in Gaussian 16 software was used to perform the quantum chemistry calculations. The theoretical DFT is calculated using the B3LYP dual-hybrid functional built-in Gaussian 16 software combined with the damping DFT-D3 dispersion correction [5,6]. The solute is geometrically optimized and frequency analyzed using the B3LYP/def2SVP computational setup to obtain an optimized structure and ensure that it is an energy minimum point. Based on frontier molecular orbital theory, B3LYP-D3(BJ)/def2TZVPP calculation level was used to obtain the energy level information of the optimized solvent. In the calculation, PCM model was used to consider the solvation effect and acetone was used as the solvent to simulate the solution environment. The electrostatic potential mapping (ESP) was acquired by the further calculation of Gaussian check files.

The binding energy (E_b) between two components was defined as follows:

$$Eb = E_{total} - E_A - E_B \quad (2)$$

Where E_{total}, E_A, and E_B are the total energy of the A–B complexes, A component, and B component, respectively [7,8].

Ab initio molecular dynamics simulations (AIMD). All the computations were conducted adopting the QUICKSTEP program as implemented in the CP2K package, which uses a mixed Gaussian and plane wave scheme with the wave functions expanded in terms of a Gaussian basis set and the charge density represented with an auxiliary plane wave basis. We employed DZVP-MOLOPT-Goedecker-Teter-Hutter (GTH) basis sets, a 600 Ry cutoff for the auxiliary plane wave expansion of the charge density, and Goedecker-Teter-Hutter (GTH) norm-conserving pseudopotentials to represent the core electrons. The calculations were conducted with the generalized gradient approximation and the Perdew-Burke-Ernzerhof (PBE)

functional. Ab initio molecular dynamics (AIMD) were used in this work. The dynamics were run with the NVT ensemble using a Nose thermostat with a damping parameter of 0.5 at a temperature of 330 K. The simulation time is 10 ps and the time step is 1.0 fs. The electrode simulation batteries were built by combining a Li-metal slab model with a 5-atom layer thick. The mentioned electrolyte was incorporated by creating a vacuum layer and placed on the surface layer, followed by solvent molecules using materials Studio amorphous packing tool. Different electrolytes and solvents were tested in this work. All AIMD simulations were run for 10 ps to analyze the reaction behaviors.

a

Item	DOL	DME
T_m (°C)	-95	-58
Viscosity (mPa s^{-1})	0.588	0.417
Dielectric constant	7.34	7.55
Donor number	21.2	21.0

b

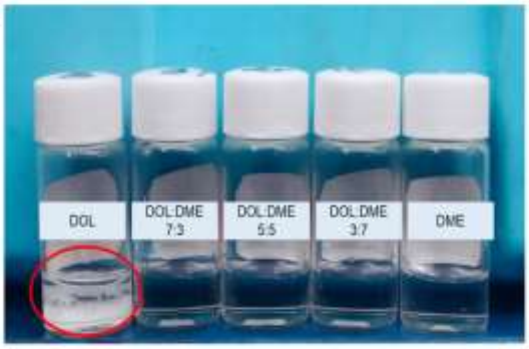

Figure S1. (a) Typical inherent properties of DOL and DME solvents. (b) The optical images of 2 M $LiBF_4$ in different electrolytes: DOL, 7DOL/3DME, 5DOL/5DME, 3DOL/7DME, and DME (v/v) (from left to right).

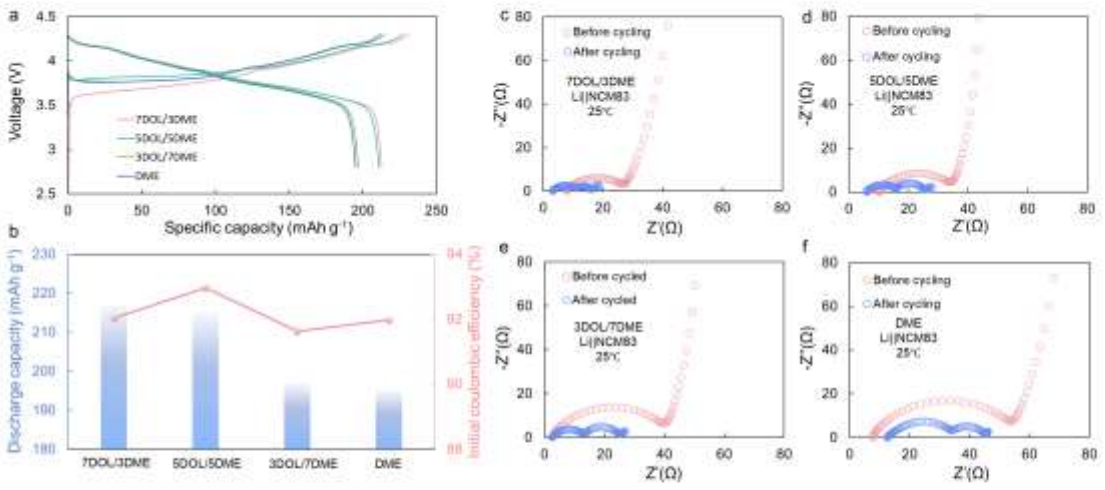

Figure S2. (a) The voltage profiles of Li||NCM83 batteries with different electrolytes at a voltage of 4.3 V under room temperature. The charge/discharge rate is 0.1 C. (b) The discharge capacity and initial Coulombic efficiency of different electrolytes based on the first charge-discharge curves at room temperature. Electrochemical impedance spectra of Li||NCM83 batteries with different electrolytes after activation at 25℃ (100% SOC) (c) 7DOL/3DME, (d) 5DOL/5DME, (e) 3DOL/7DME, and (f) DME. The loading of active material is 9.0~10.0 mg cm^{-2}.

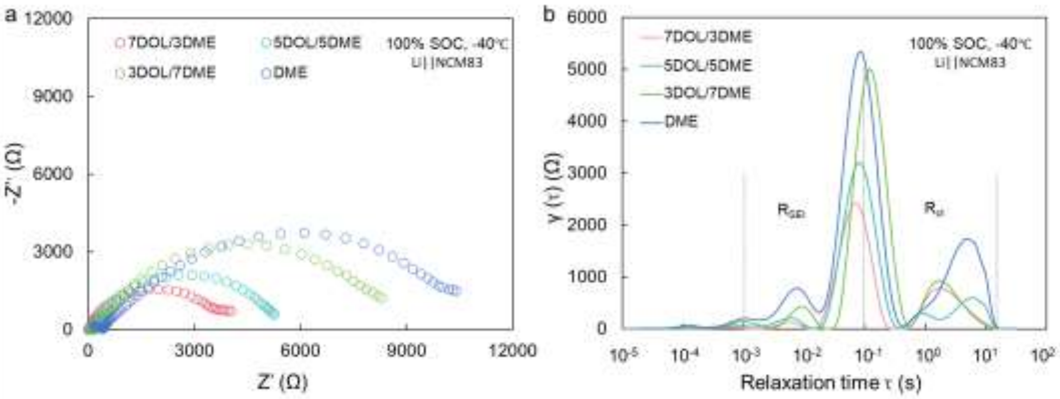

Figure S3. (a) EIS measurements for Li||NCM83 batteries with different electrolytes. (b) DRT plot derived from EIS data. The batteries were activated at room temperature to a fully charged state (100% SOC), followed by electrochemical characterization at −40°C.

Note : (Supplementary Figure 1-3) We prepared five ether-based electrolytes with 2 M $LiBF_4$ in different solvent ratios: DOL, 7DOL/3DME, 5DOL/5DME, 3DOL/7DME, and DME (v/v). **Figure S1** presents the optical images of these electrolytes. Notably, the use of DOL alone is impractical for low-temperature battery applications due to its tendency to polymerize and solidify in the presence of inorganic salts. We then evaluated the compatibility of the remaining electrolytes (7DOL/3DME, 5DOL/5DME, 3DOL/7DME, and DME) with Li||NCM83 batteries (**Figure S2**). The EIS measurements were carried out before and after room-temperature activation to analyze the kinetics of different interfacial processes. The increase in the proportion of DME leads to an elevated interfacial resistance, and the ohmic resistance also changes. Following room-temperature activation at 100% state of charge (SOC), the battery was set at –40°C for 4 to 6 hours. Impedance measurements were subsequently conducted, and the data were converted for distribution of relaxation times (DRT) analysis (**Figure S3**). The results indicate that as the proportion of DME increases, the interfacial charge-transfer resistance and ionic transport impedance rise significantly. This increase is primarily attributed to the strong binding energy between DME and Li^+ , which hinders the desolvation. Additionally, DME tends to form solvent-derived organic interphases that impede rapid ionic transport. **Thus**, the **7DOL/3DME** ratio can simultaneously enhance the low-temperature stability and interfacial dynamics of the electrolyte while ensuring sufficient ionic conductivity.

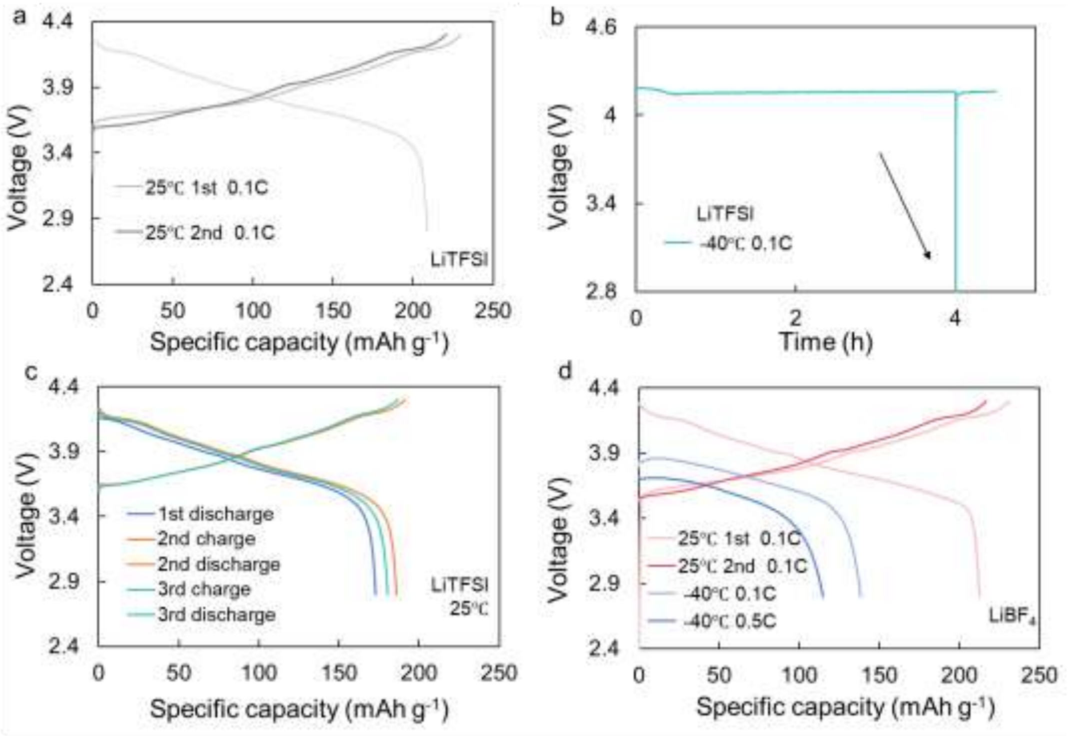

Figure S4. (a) Galvanostatic charge/discharge voltage profiles of the Li|LiTFSI|NCM83 battery at room-temperature (25℃). (b) The voltage-time profiles of LiTFSI electrolyte at –40℃. (c) Room-temperature (25℃) charge/discharge curves of Li|LiTFSI|NCM83 battery after battery failure at –40℃. (d) The galvanostatic charge/discharge voltage profiles of the Li|$LiBF_4$|NCM83 battery at 25℃ and –40℃.

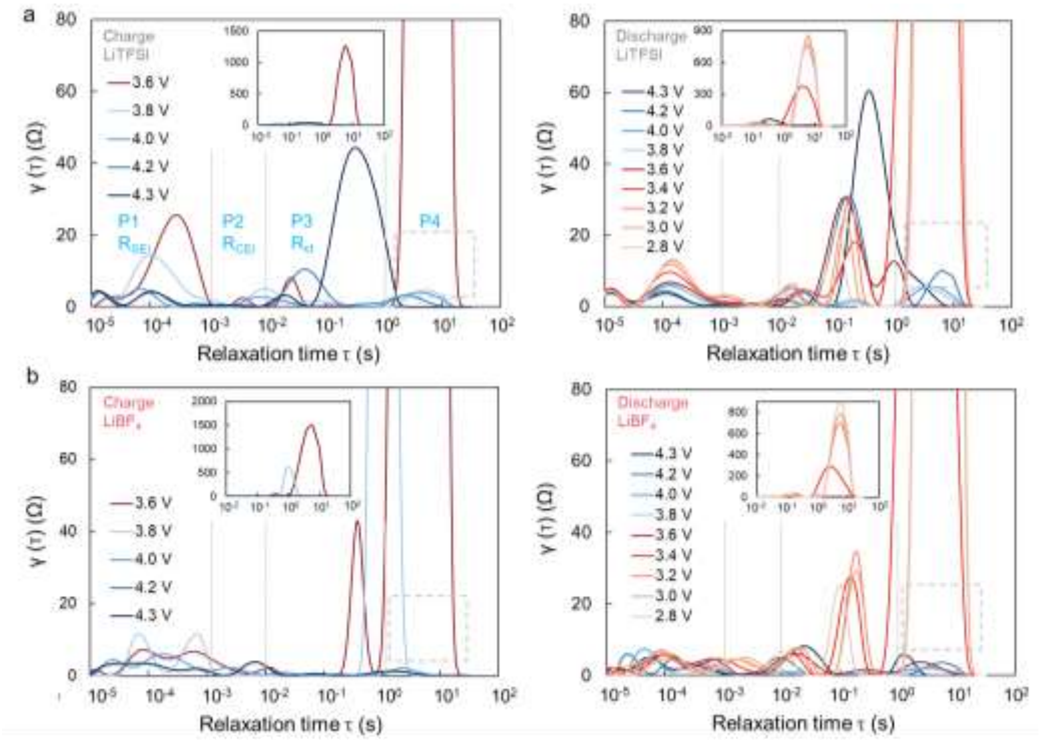

Figure S5. DRT plot derived from in situ EIS data in (a) Li|LiTFSI|NCM83 batteries and (b) Li|$LiBF_4$|NCM83 batteries during the first cycle.

Note: The peaks with relaxation times in the ranges of 10^{-4}-10^{-3} s, 10^{-3}-10^{-2} s, 10^{-2}-10^{0} s, and 10^{0}-10^{1} s account for the growth process of the SEI, CEI, the charge transfer process, as well as the diffusion process of lithium ions, respectively [9-11].

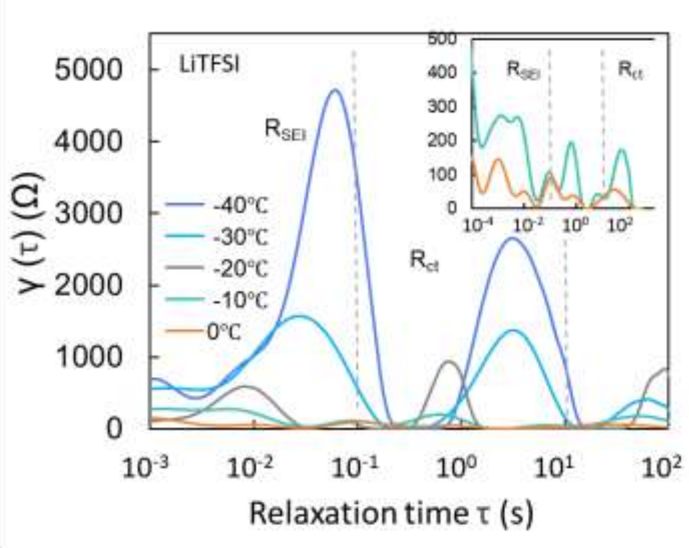

Figure S6. Temperature-dependent DRT plot derived from EIS data in Li||NCM83 batteries for LiTFSI-based electrolyte. (The DRT profiles of Li||NCM83 batteries were activated at room temperature to a fully charged state (100% SOC) and subsequently measured across a temperature range from 0℃ to –40℃)

Note: As shown in Figures 1g and S6, the values of R_{ct} located at the frequency (10^{1}> τ >10^{-1}), correspond to the kinetics of Faradic processes occurring cathodes related to the Li^{+} (de)solvation. The intermediate frequency peak (10^{-1}> τ >10^{-3}) can be assigned to the impedance of SEI (R_{SEI}). Some studies attribute this to SEI-dominated interfacial processes while neglecting the role of the CEI, presumably due to the thinner CEI film [11-13] [14].

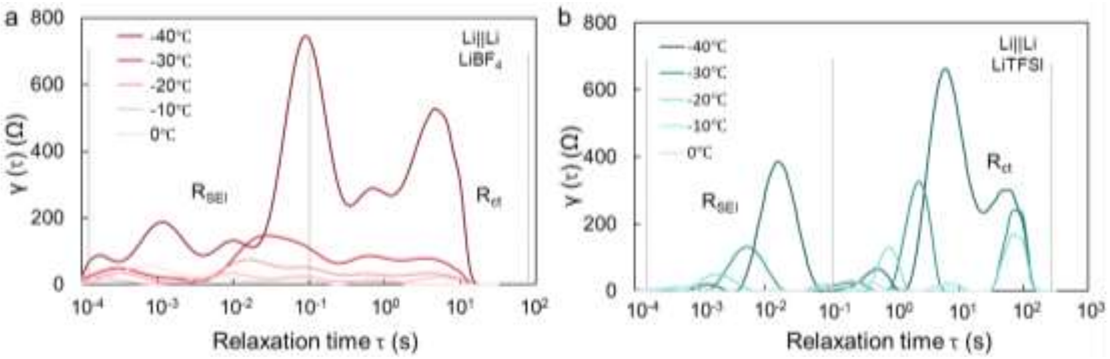

Figure S7. Temperature-dependent distribution of DRT plot derived from EIS data for (b) Li| $LiBF_4$|Li and (c) Li| LiTFSI |Li.

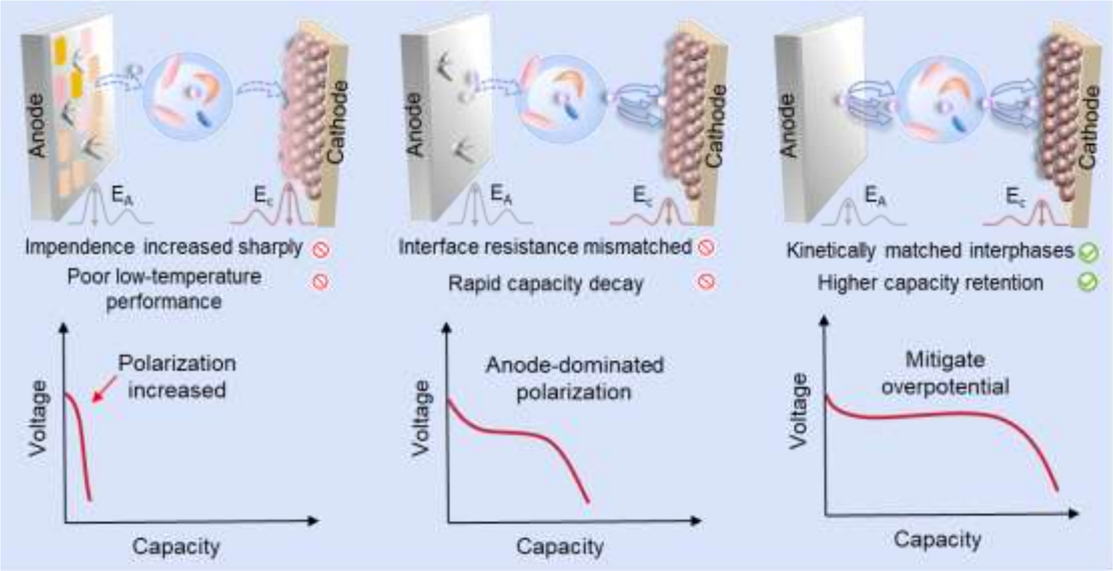

Figure S8. Proposed low-temperature electrolyte design principle.

Note: From a kinetic perspective, low temperatures can lead to elevated battery polarization, resulting in voltage loss. Battery polarization encompasses ohmic polarization, concentration polarization, and electrochemical polarization. Consequently, the working voltage (E) of the battery during discharge process adheres to the following equation [15,16]:

$$E = E_0 - [(\eta_{ct})_a + (\eta_c)_a] - [(\eta_{ct})_c + (\eta_c)_c] - IR_i \quad (3)$$

Where E_0 refers to open-circuit voltage, $(\eta_{ct})_a$ and $(\eta_{ct})_c$ represent the active/electrochemical-polarization or ohmic-polarization overpotential of the anode and cathode, respectively. $(\eta_c)_a$ and $(\eta_c)_c$ correspond to concentration-polarization overpotential at the anode and cathode. I is the work current, and R_i is the internal resistance of the battery. This equation shows that the battery voltage is influenced by polarization and ohmic resistance at both the anode and cathode. These factors are both subject to limitations imposed by temperature. Given that the low-temperature discharge polarization mainly arises from electrochemical polarization, specifically the charge-transfer kinetics at the interface, rather than from mass transport in the electrolyte and electrodes. In this context, we explored several potential scenarios to systematically analyze the synergistic effects of the CEI and SEI during the discharge

process. Based on a typical Arrhenius-type relation [17], the values of E_C ($E_{Cathode}$) and E_A (E_{Anode}) determine the charge transfer resistance R_{ct} measured during the discharging process. R_{ct} refers to Li^+ (de)solvation and transfer across multiple phase boundaries, which has long been energy-consuming [18].

$$\frac{1}{R_{ct}} = A_0 e^{-E/RT}$$

Where A_0, R, and E stand for the pre-exponential constant, the standard gas constant, and the charge transfer activation energy, respectively.

In one case, high E_C and E_A at both electrodes lead to a significant capacity decline due to large polarization. In contrast, a smaller battery polarization in another scenario is expected to prolong the constant current stage and improve capacity, owing to the decrease of E_C. However, the mismatch in the kinetics of the cathode and anode exacerbates polarization, particularly under elevated current densities. The sluggish kinetics contribute to detrimental side reactions at the interface. These include the growth of lithium dendrites and the subsequent formation of SEI, posing a significant challenge for developing next-generation high-power-density LMB technologies [12,19].

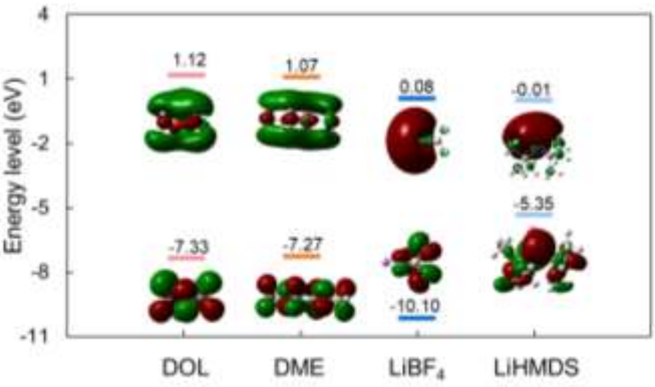

Figure S9. HOMO and LUMO energy levels of DOL, DME, $LiBF_4$, and LiHMDS.

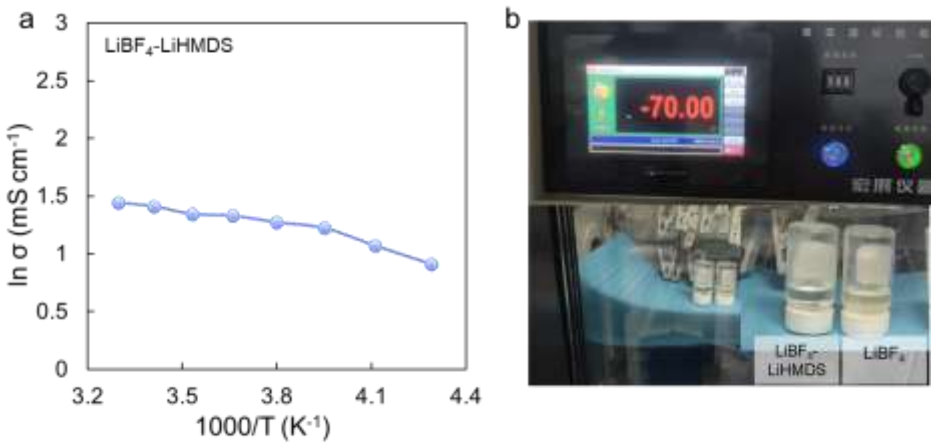

Figure S10. (a) Temperature dependence of the ionic conductivity of the $LiBF_4$-LiHMDS electrolyte. (b) Photographs of the $LiBF_4$ and $LiBF_4$-LiHMDS electrolytes when stored at −70℃.

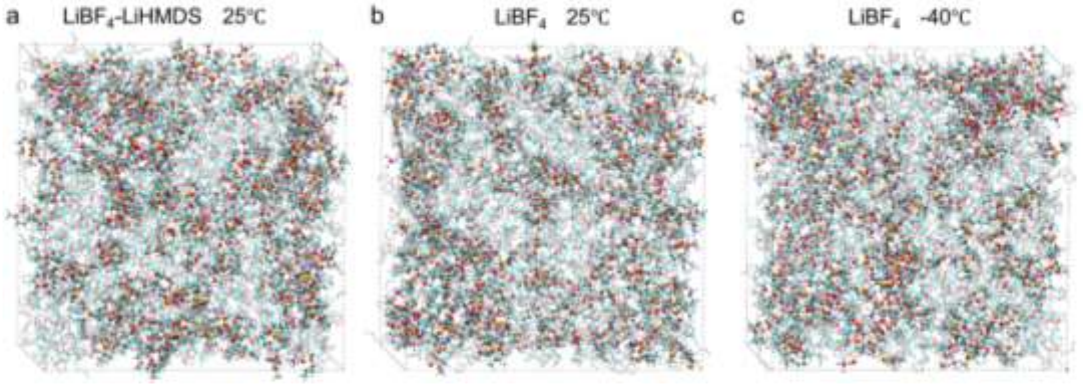

Figure S11. Snapshots for distributions of anions and solvents in (a) $LiBF_4$-LiHMDS electrolyte at 25℃ (b) $LiBF_4$ electrolyte at 25℃ and (c) $LiBF_4$ electrolyte at –40℃.

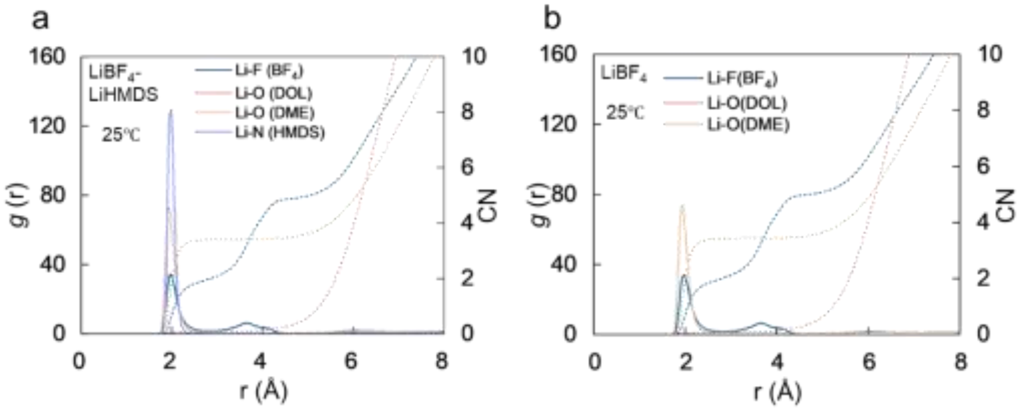

Figure S12. RDF of different components at 25℃ in (a) $LiBF_4$-LiHMDS (b) $LiBF_4$-based electrolyte.

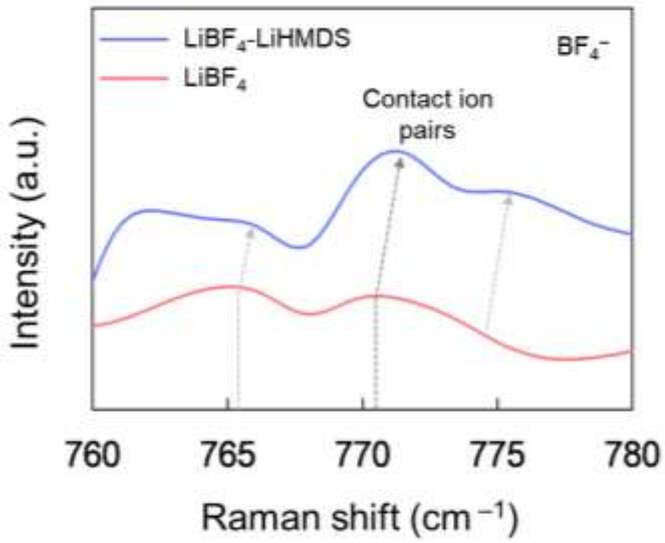

Figure S13. Raman spectra of BF_4^- anions with $LiBF_4$-LiHMDS and $LiBF_4$-based electrolyte.

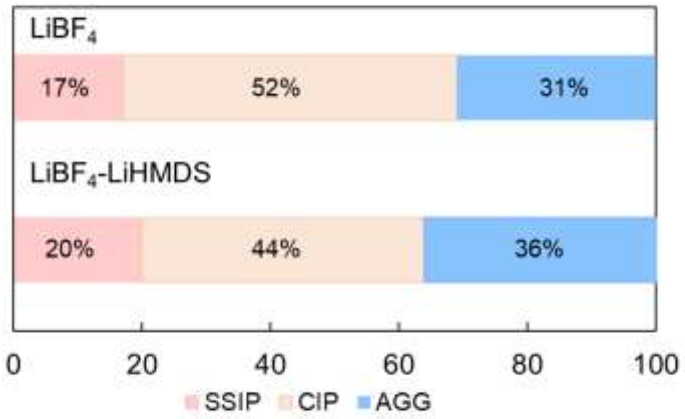

Figure S14. The distributions of the solvates in the $LiBF_4$-based and $LiBF_4$-LiHMDS electrolytes extracted from the MD simulation trajectories.

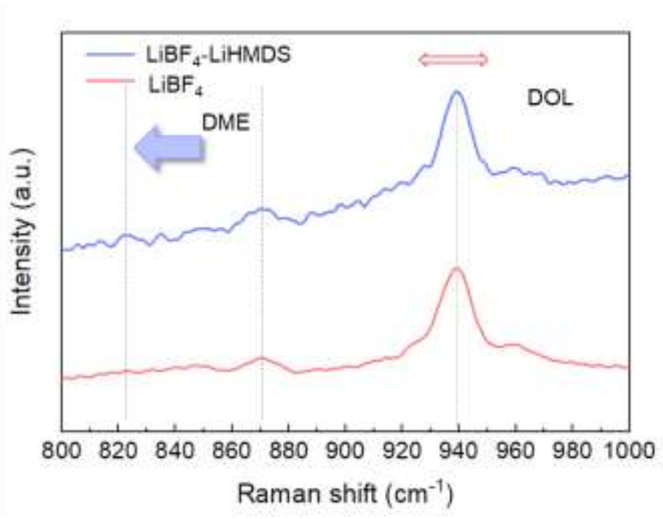

Figure S15. Raman spectra of DOL and DME solvents with $LiBF_4$-LiHMDS and $LiBF_4$-based electrolyte.

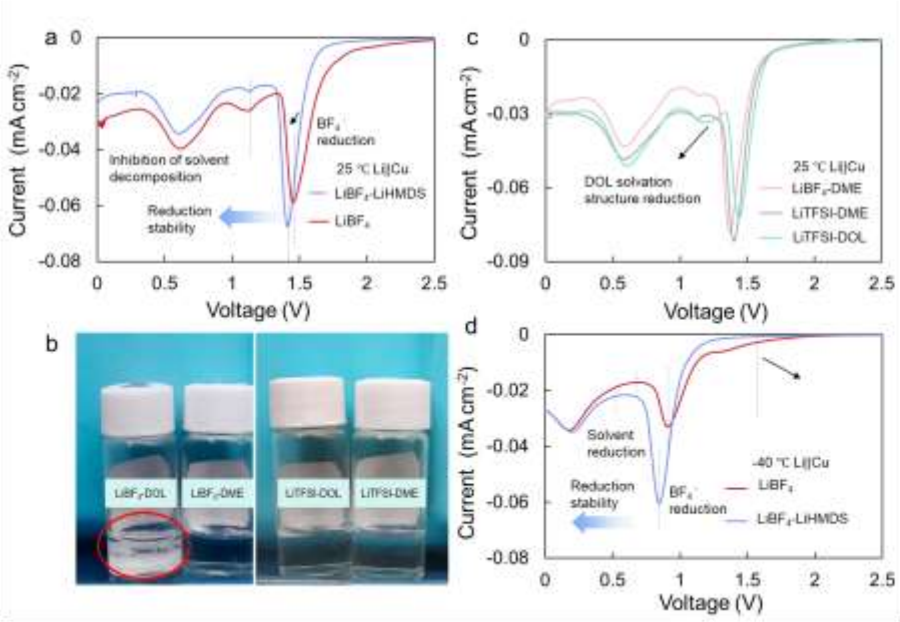

Figure S16. (a) LSV curves of Li || Cu batteries for measurement of reduction potential in voltage range of 0-2.5 V with different electrolytes under (a) (c) 25℃ and (d) –40℃. The scan rate is 1 mV s^{-1}. (b) The optical images of 2 M $LiBF_4$ in DOL, 2 M $LiBF_4$ in DME, 2 M LiTFSI in DOL, and 2 M LiTFSI in DME (from left to right).

Note: As shown in Figure S16 a, a clear broad peak appeared between 1.00-1.30 V. To further elucidate the source of this broad peak, we prepared electrolytes with different salts and solvents and tested LSV under same conditions. It should be noted that due to the hydrolysis of DOL and $LiBF_4$, we were could not obtain the electrolyte solution (Figure S16 b). Instead, we proceeded with experiments using a 2 M LiTFSI-DOL electrolyte. The results show a broad peak between 1.3-0.9 V for the 2 M LiTFSI-DOL electrolyte prepared with DOL alone. In contrast, electrolytes with DME as the solvent (2 M $LiBF_4$-DME and 2 M LiTFSI-DME) did not exhibit a clear broad peak, only showing a similar peak at 1.10 V, which may be attributed to the decomposition of the DOL solvation structure (Figure S16 c). According to relevant literature, the peak near 0.5 V may be attributed to the decomposition of free solvent DOL or DME [20,21]. When LiHMDS was introduced, the reduction stability of the electrolyte was improved.

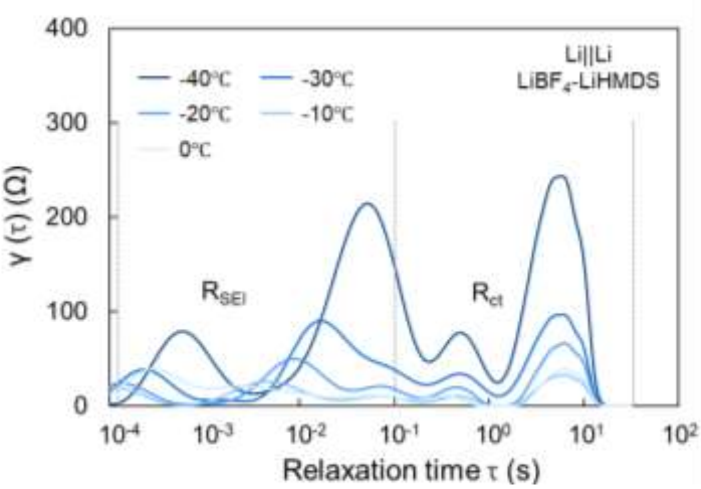

Figure S17. Temperature-dependent distribution of DRT plot derived from EIS data for Li|$LiBF_4$-LiHMDS|Li.

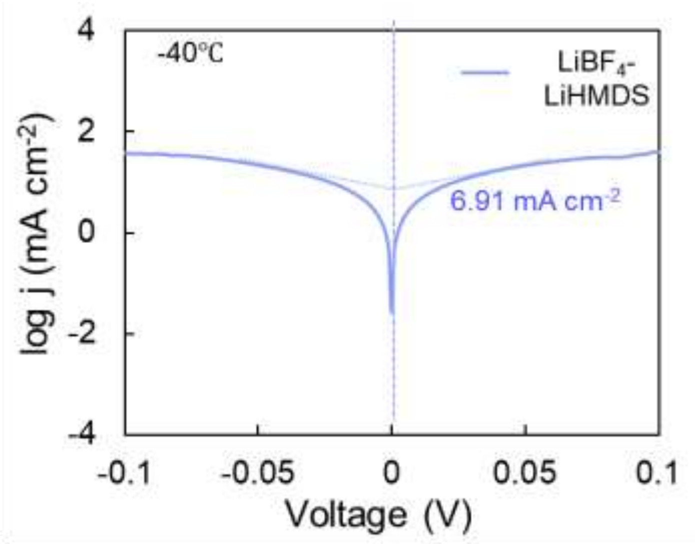

Figure S18. Tafel plot of Li plating/stripping in $LiBF_4$-LiHMDS electrolytes at -40℃.

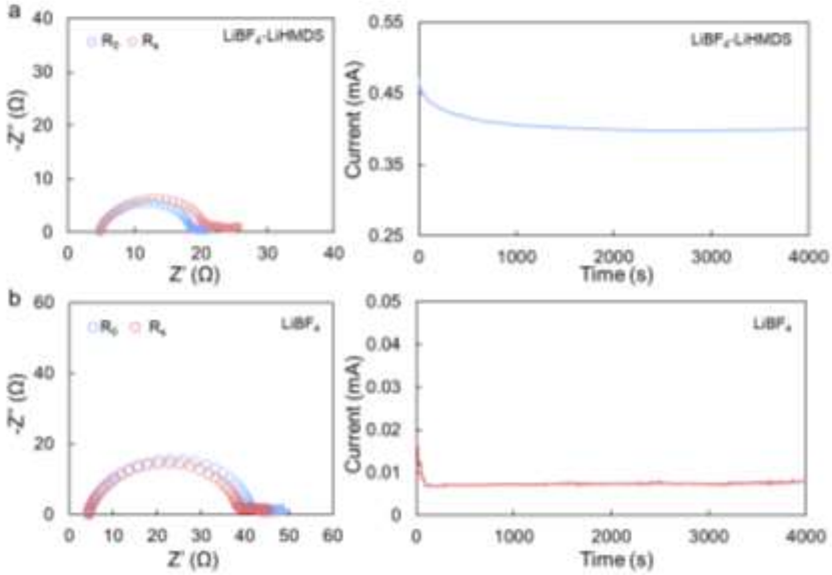

Figure S19. Selected data for transference number calculations in coin batteries. 10 mV polarization curves and complex impedance plots before and after polarization used for t_{Li}^+ calculations of (a) $LiBF_4$-LiHMDS and (b) $LiBF_4$ electrolyte.

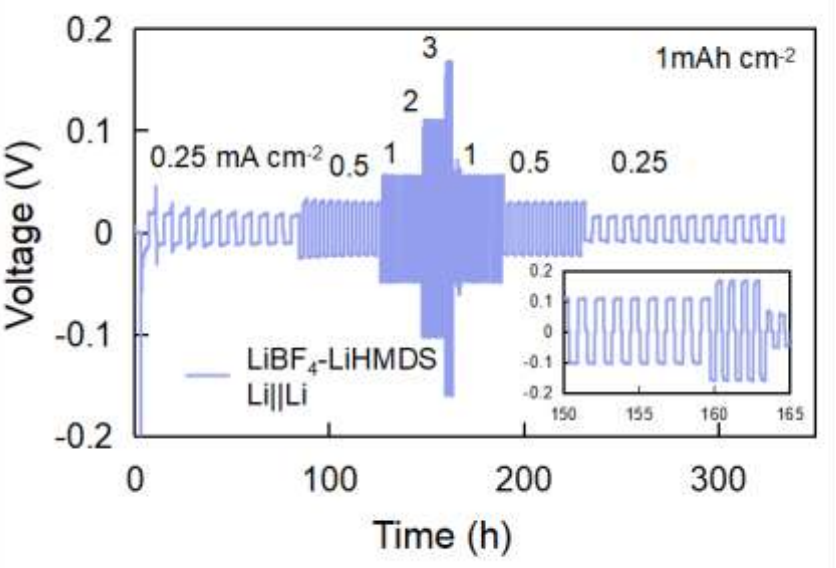

Figure S20. Rate performance of Li|LiBF$_4$-LiHMDS|Li symmetric coin batteries at current densities from 0.25 to 3 mA cm^{-2} at -40 ℃.

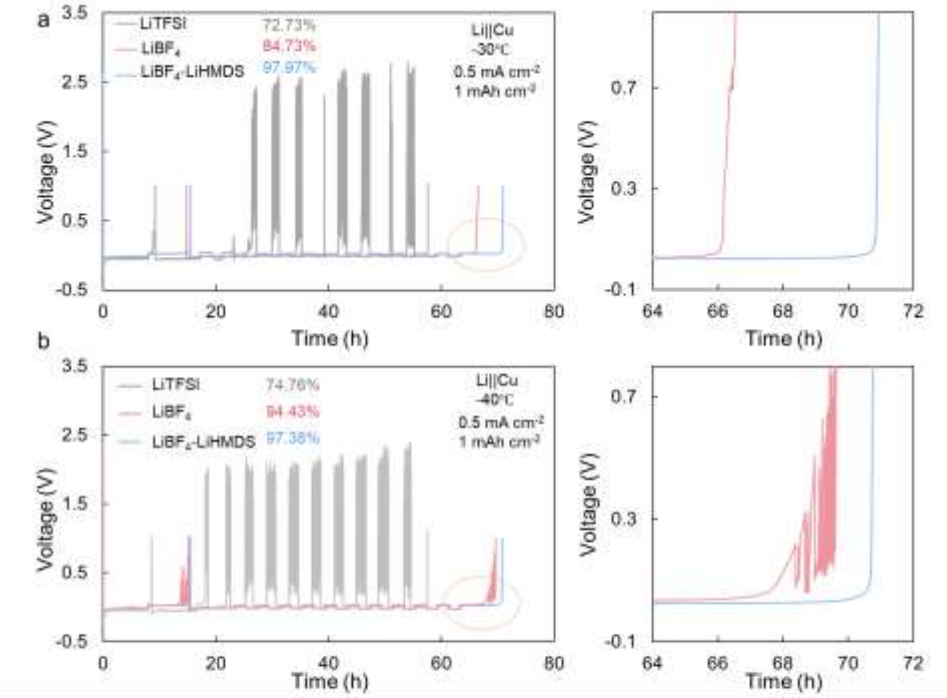

Figure S21. Measurement of CE of Li plating/stripping in Li||Cu batteries using a modified Aurbach method at (a) –30℃ and (b) –40℃. (Inset: measurement of CE from 64 h to 72 h)

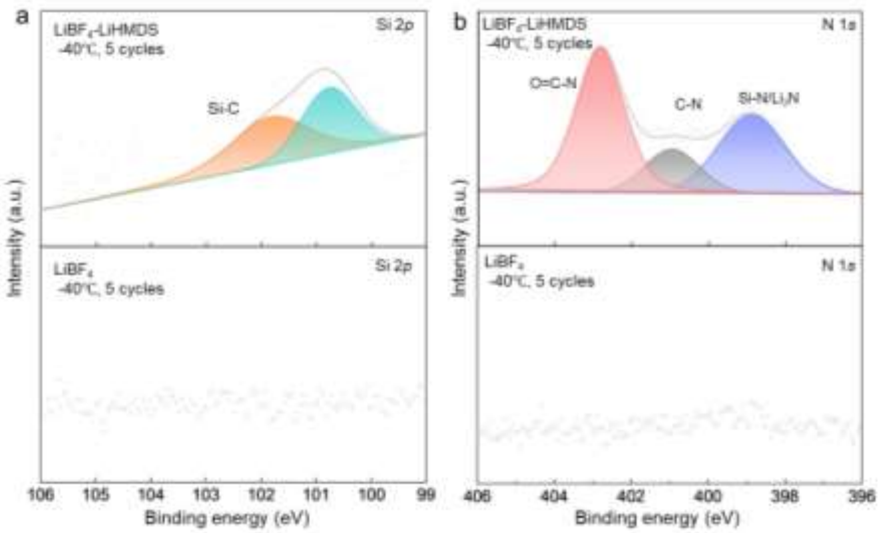

Figure S22. XPS analysis of (a) Si 2*p* and (b) N 1*s* for cycled anodes at –40℃ in $LiBF_4$-LiHMDS electrolytes. The batteries were disassembled in the fully discharged state at –40℃.

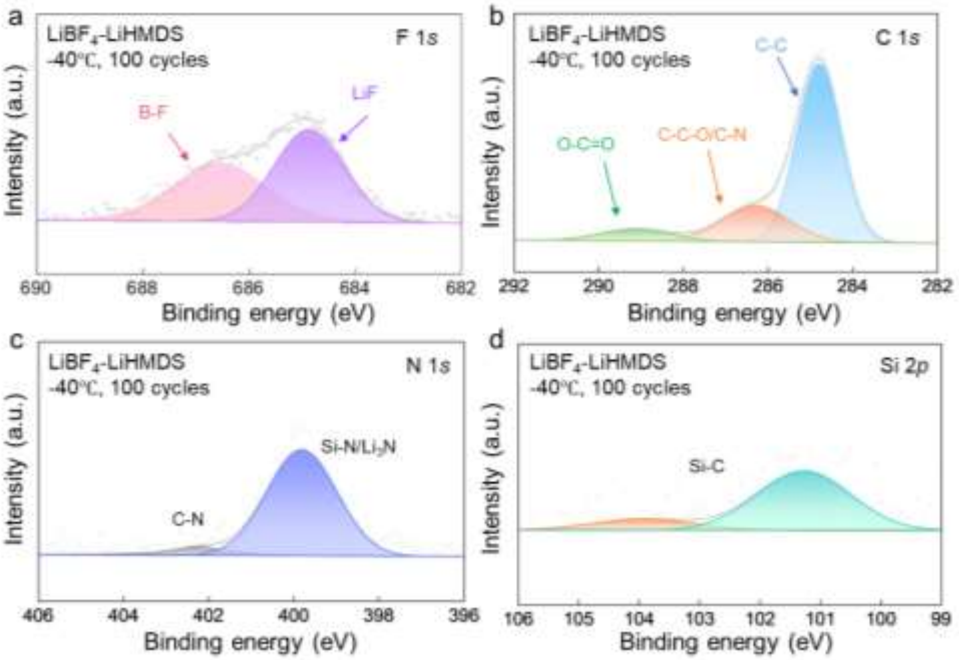

Figure S23. XPS analysis of (a) F 1*s* (b) C 1*s* (c) N 1*s* (d) Si 2*p* of $LiBF_4$-LiHMDS electrolytes after 100 cycles. The batteries were cycled at –40℃ and disassembled in the fully discharged state.

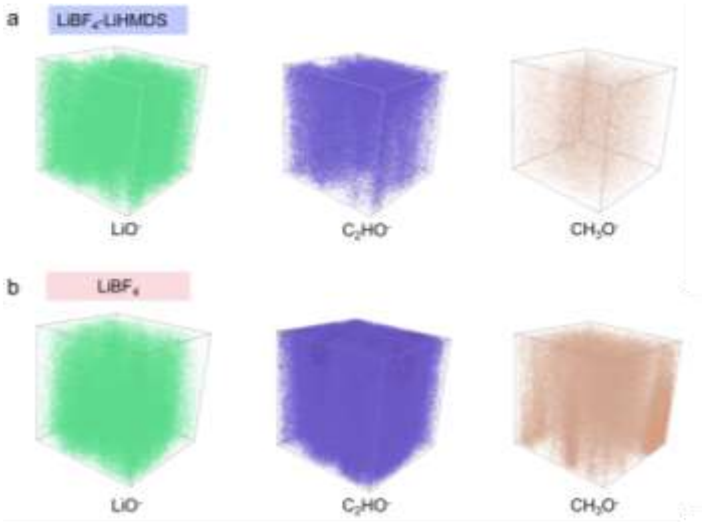

Figure S24. TOF-SIMS three-dimensional depth-profiling images of the LiO^{-}, C_2HO^{-}, and CH_3O^{-} species in the SEIs formed in the (a) $LiBF_4$-LiHMDS and (b) $LiBF_4$ electrolytes. The interphases were formed at room temperature and cycled at 0.1 C under –40℃.

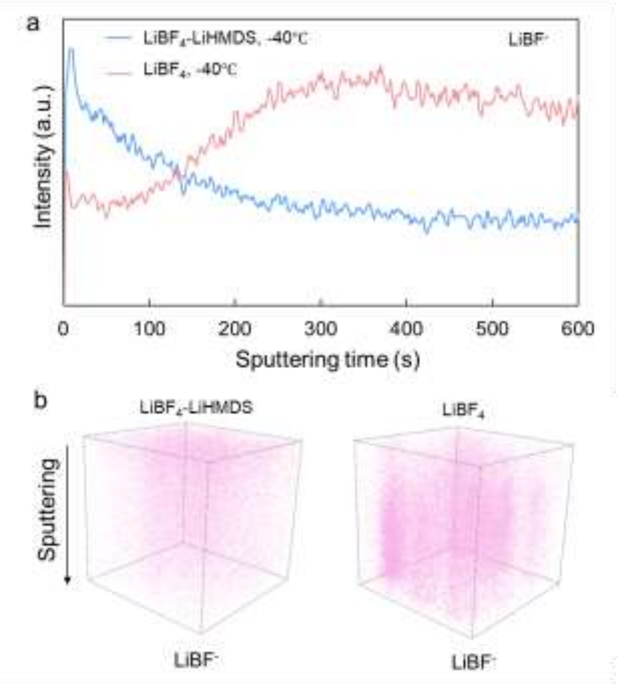

Figure S25. (a) Depth profiles of secondary ion fragments ($LiBF^-$) obtained by TOF-SIMS analysis of the SEIs. TOF-SIMS three-dimensional images of the $LiBF^-$ species in the SEIs formed in the (b) $LiBF_4$-LiHMDS and $LiBF_4$ electrolytes. The interphases were formed at room temperature and cycled at 0.1 C under –40℃.

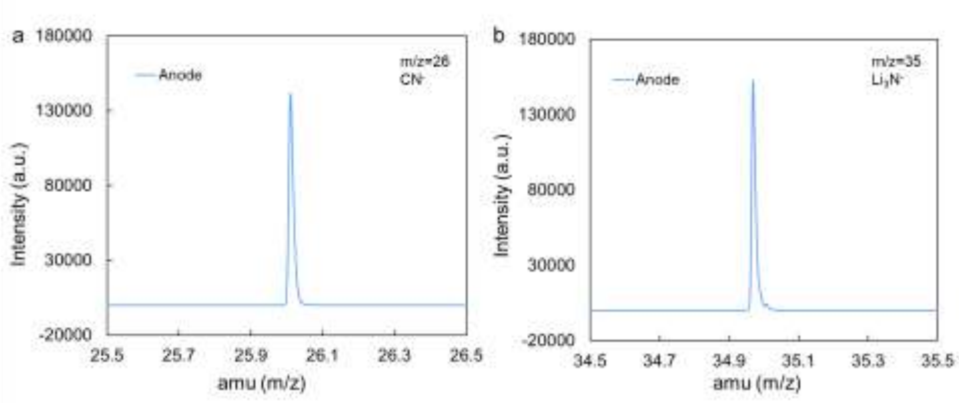

Figure S26. TOF-SIMS analysis of lithium metal anode after cycling in $LiBF_4$-LiHMDS electrolyte at –40 ℃ (a) $CN^−$ (b) $Li_3N^−$. The battery was disassembled at a fully discharged state.

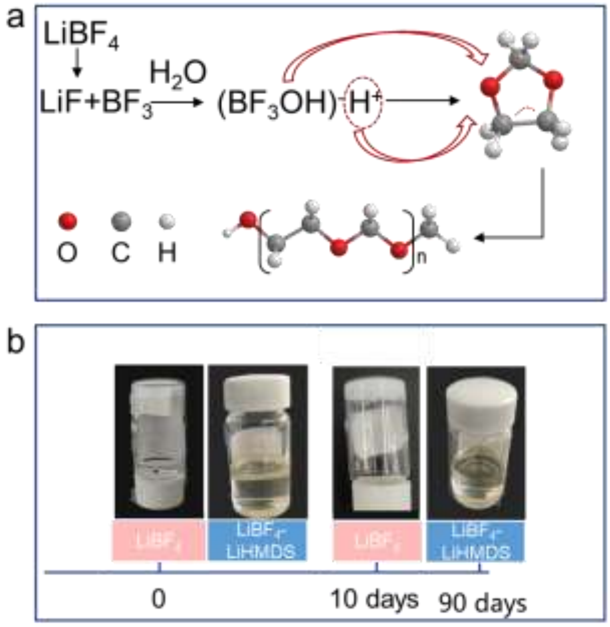

Figure S27. (a) Schematic diagram showing the proton initiation mechanism for the ring-opening polymerization process of DOL monomer. (b) Optical photographs of various electrolyte solutions after storage.

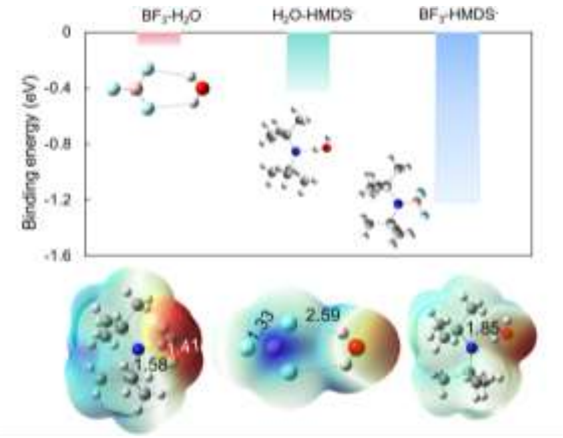

Figure S28. Bond length and binding energy of BF_3-H_2O, H_2O-$HMDS^-$. and BF_3-$HMDS^-$ (Data with the bond length in Å).

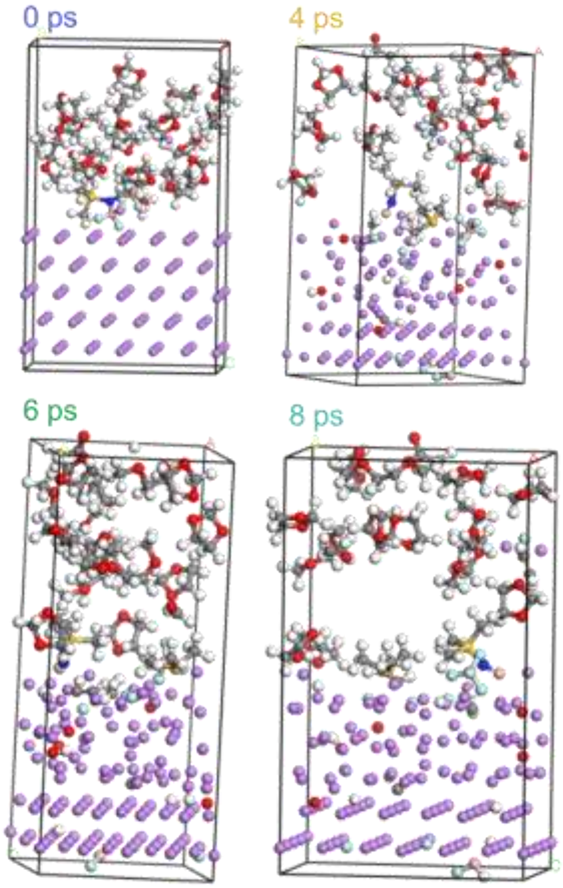

Figure S29. Snapshots of 0 ps, 4 ps, 6 ps, and 8 ps AIMD simulation for the adsorption and decomposition of $LiBF_4$-LiHMDS solvation complex on the Li surface. In the ball-and-stick representation, the atoms are represented by H: white, C: gray, O: red, Si: yellow, Li: purple, N: blue, F: cyan, B: pink.

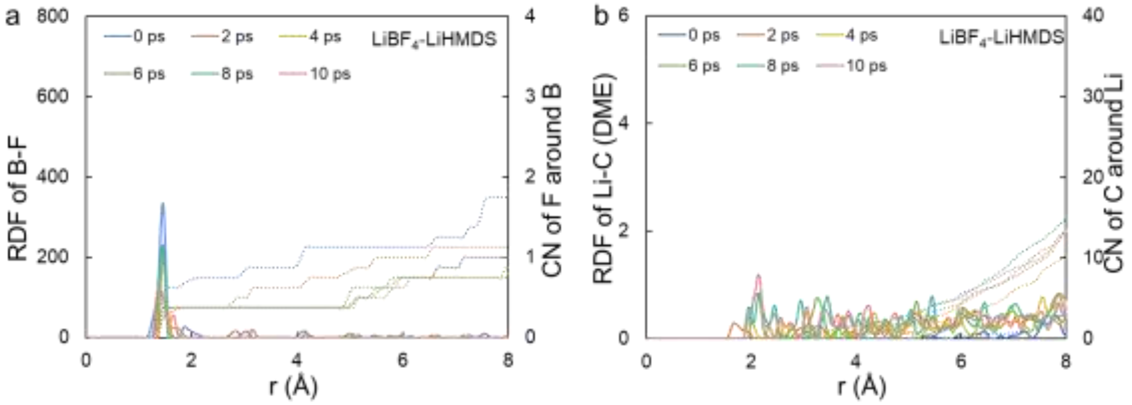

Figure S30. RDF evolution during the simulation process with $LiBF_4$-LiHMDS electrolytes. (a) B-F (b) Li-C (DME)

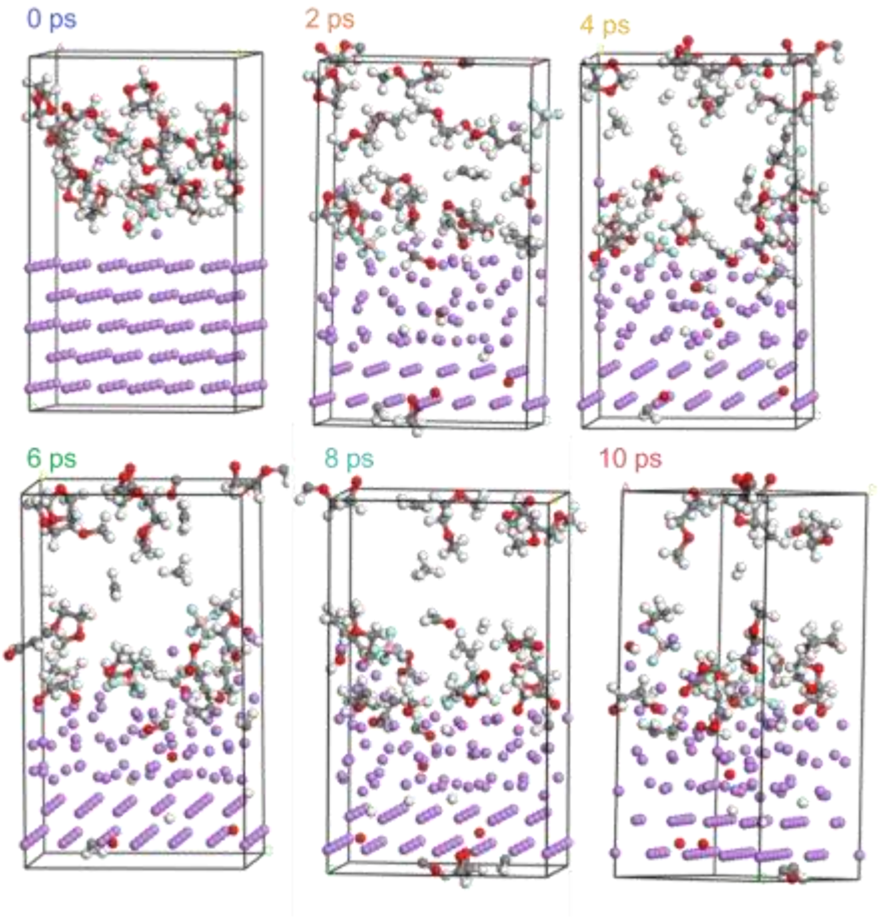

Figure S31. Snapshots of 0 ps, 2 ps, 4 ps, 6 ps, 8 ps, and 10 ps AIMD simulation for the adsorption and decomposition of $LiBF_4$-based solvation complex on the Li surface. In the ball-and-stick representation, the atoms are represented by H: white, C: gray, O: red, Si: yellow, Li: purple, N: blue, F: cyan, B: pink.

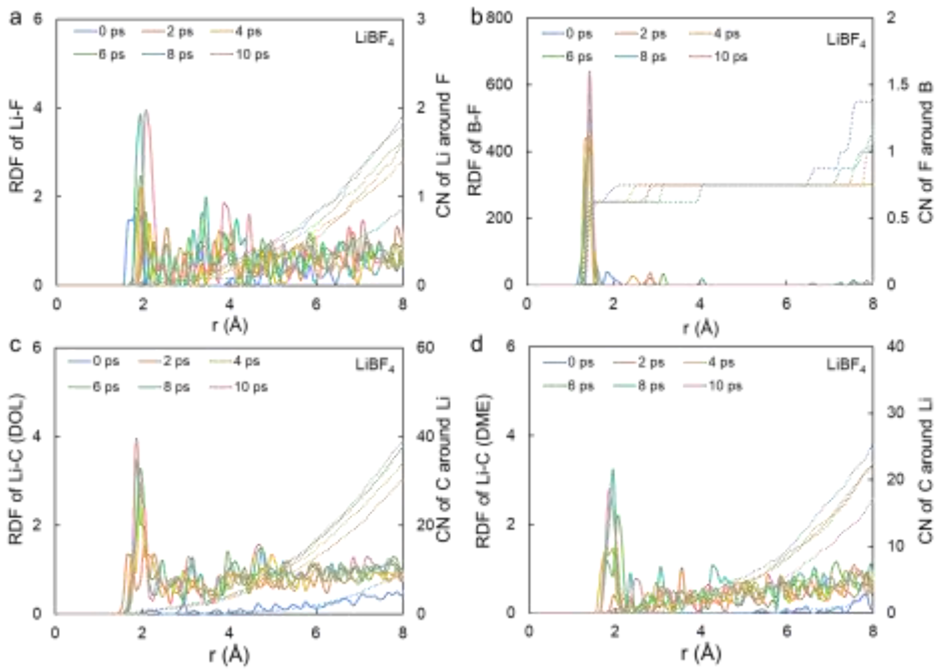

Figure S32. RDF evolution during the simulation process with $LiBF_4$-based electrolytes. (a) Li-F (c) B-F (b) Li-C (DOL) and (d) Li-C(DME)

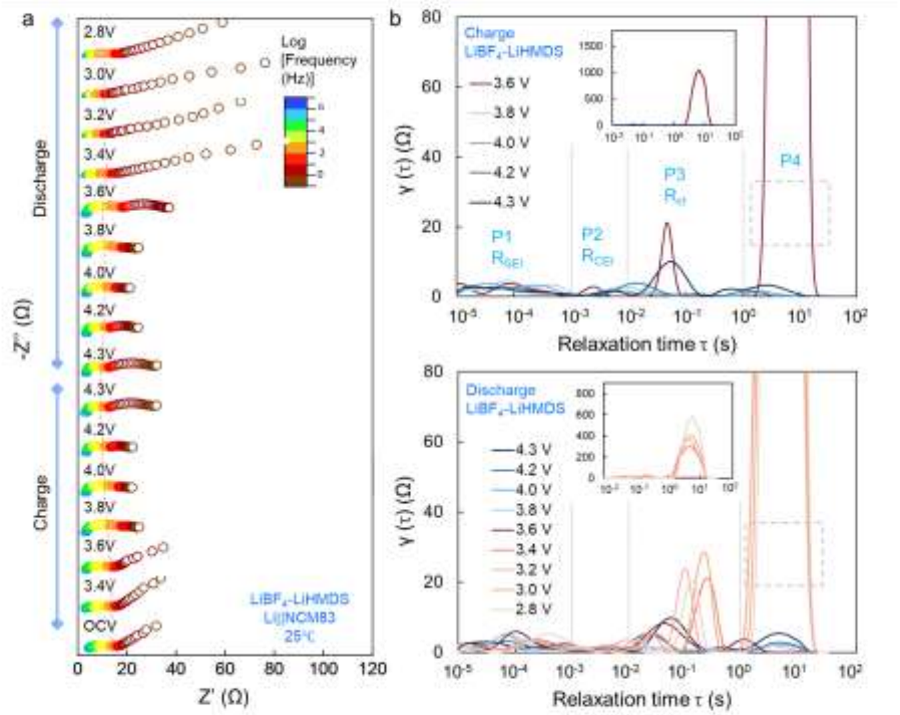

Figure S33. (a) In situ EIS and (b) DRT plot derived from in situ EIS data of Li||NCM83 batteries during the first cycle at room temperature in $LiBF_4$-LiHMDS electrolyte.

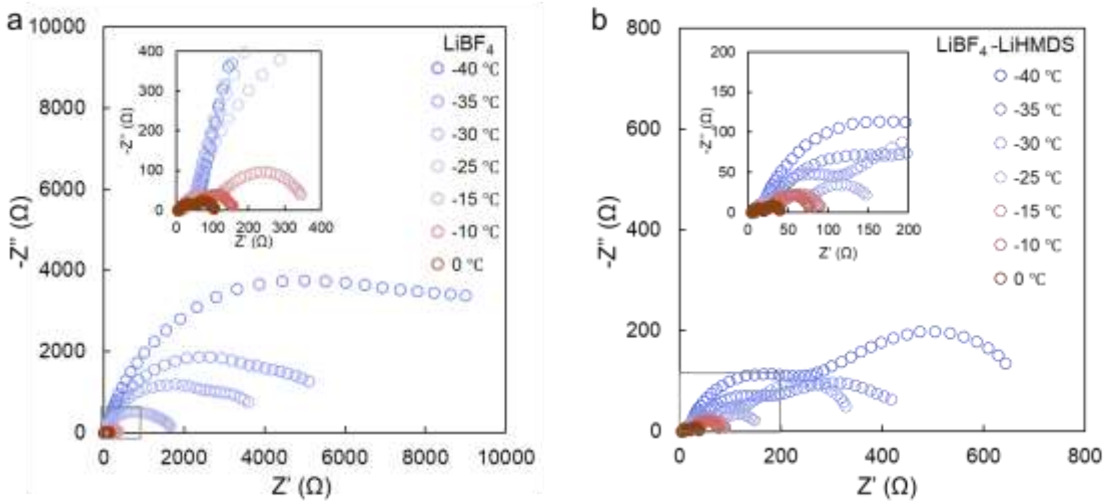

Figure S34. Nyquist plots of Li||NCM83 at various temperatures in (a) $LiBF_4$ and (b) $LiBF_4$-LiHMDS electrolytes.

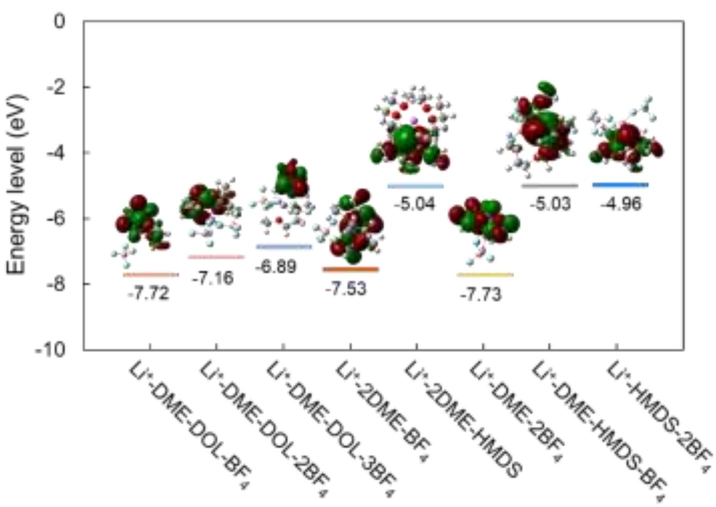

Figure S35. HOMO energy of representative solvation configurations.

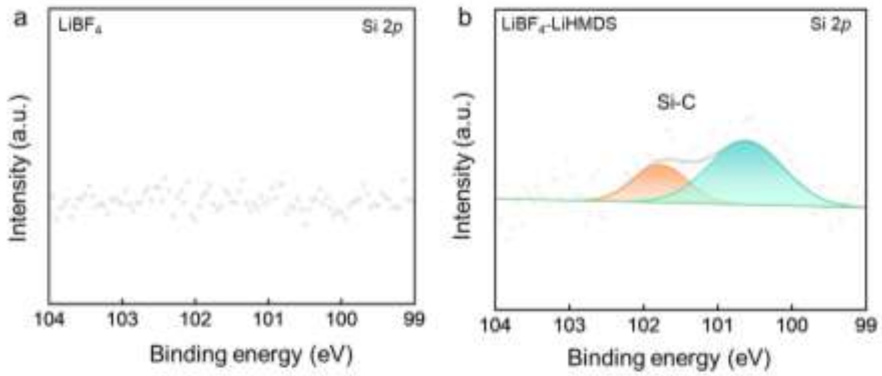

Figure S36. XPS analysis of Si 2*p* for cycled NCM83 cathodes at –40 ℃ in (a) $LiBF_4$ and (b) $LiBF_4$-LiHMDS electrolytes.

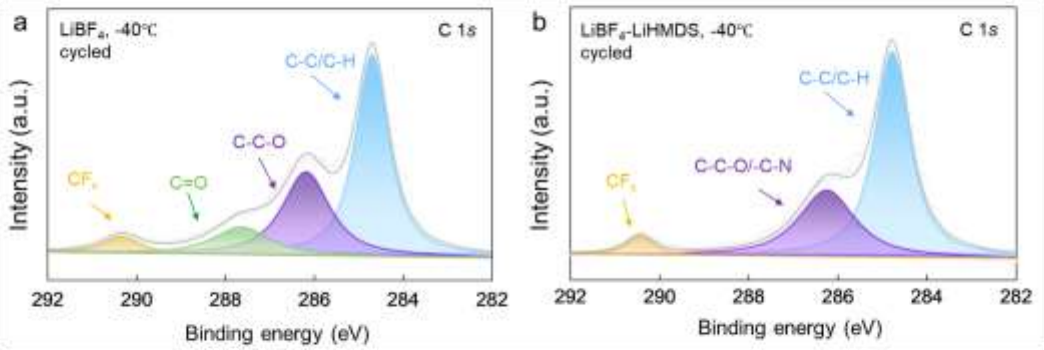

Figure S37. XPS analysis of C 1*s* for cycled NCM83 cathodes in (a) $LiBF_4$ and (b) $LiBF_4$-LiHMDS electrolytes.

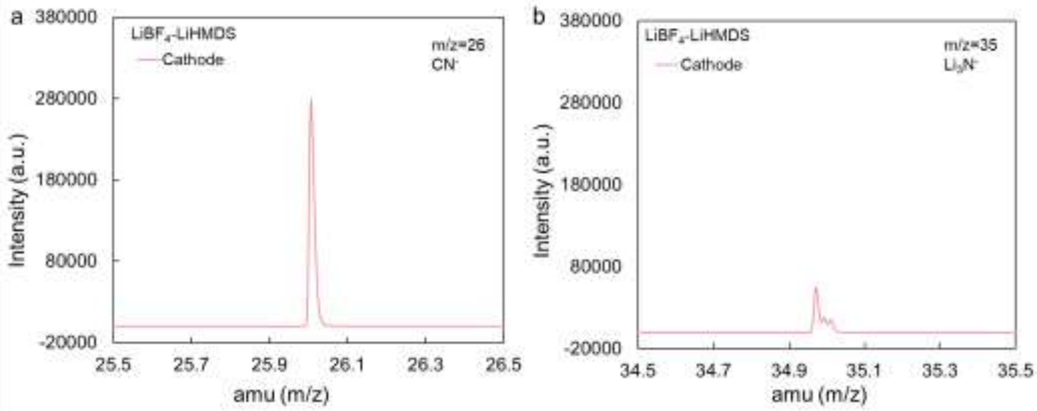

Figure S38. TOF-SIMS analysis of NCM83 cathode after cycling at –40 ℃ (a) CN^{-} (b) Li_3N^{-}. The battery was disassembled at a fully discharged state.

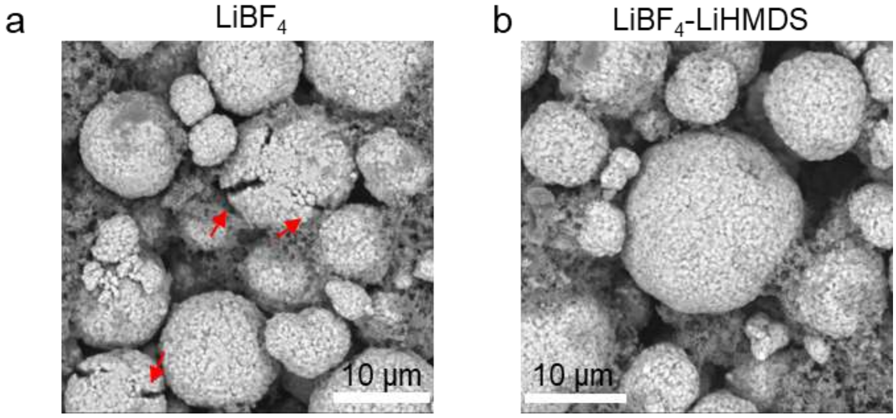

Figure S39. SEM morphology of cycled at –40 ℃ cathodes in (a) $LiBF_4$ and (b) $LiBF_4$-LiHMDS electrolytes.

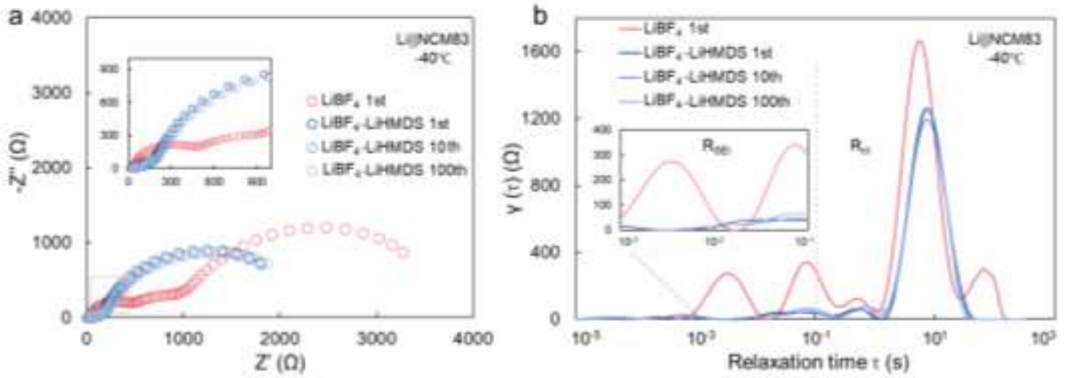

Figure S40. (a) EIS measurements for Li||NCM83 batteries after different charge/discharge cycles with $LiBF_4$ and $LiBF_4$-LiHMDS electrolytes. (b) DRT plot derived from EIS data. The tests were conducted at –40℃ in the fully discharged state.

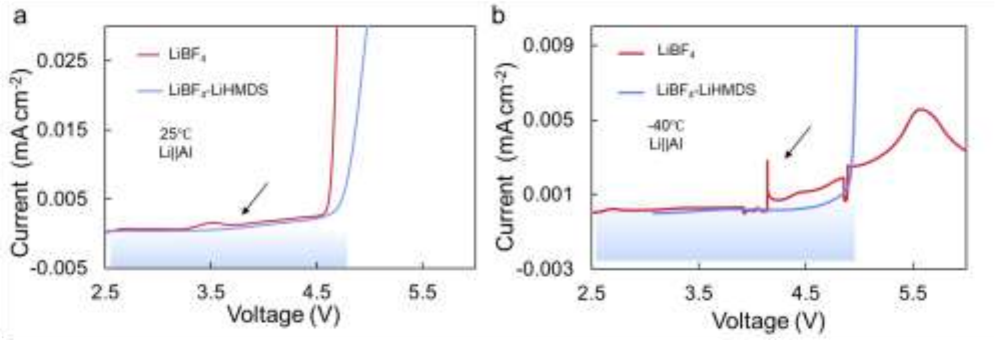

Figure S41. LSV curves of Li || Al batteries for measurement of oxidation potential in 2.5-6.0 V under (a) 25℃ and (b) –40℃ with $LiBF_4$ and $LiBF_4$-LiHMDS electrolytes (The scan rate is 0.5 mV s^{-1}).

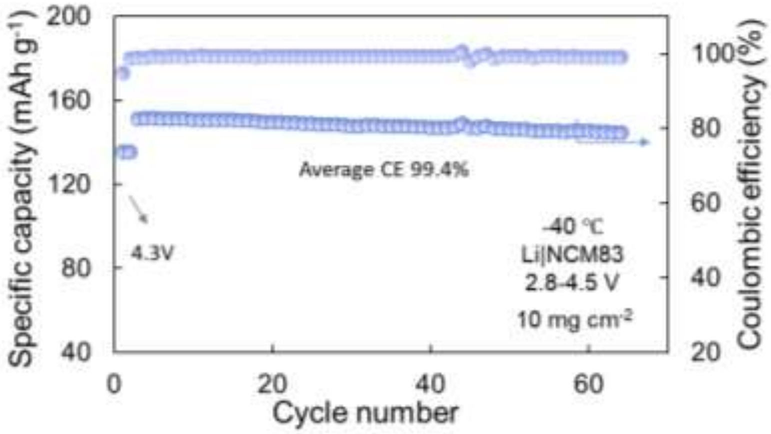

Figure S42. Cycling performance of 4.5 V Li||NCM83 using $LiBF_4$-LiHMDS at −40 ℃ with a charge/discharge rate of 0.2/0.2 C.

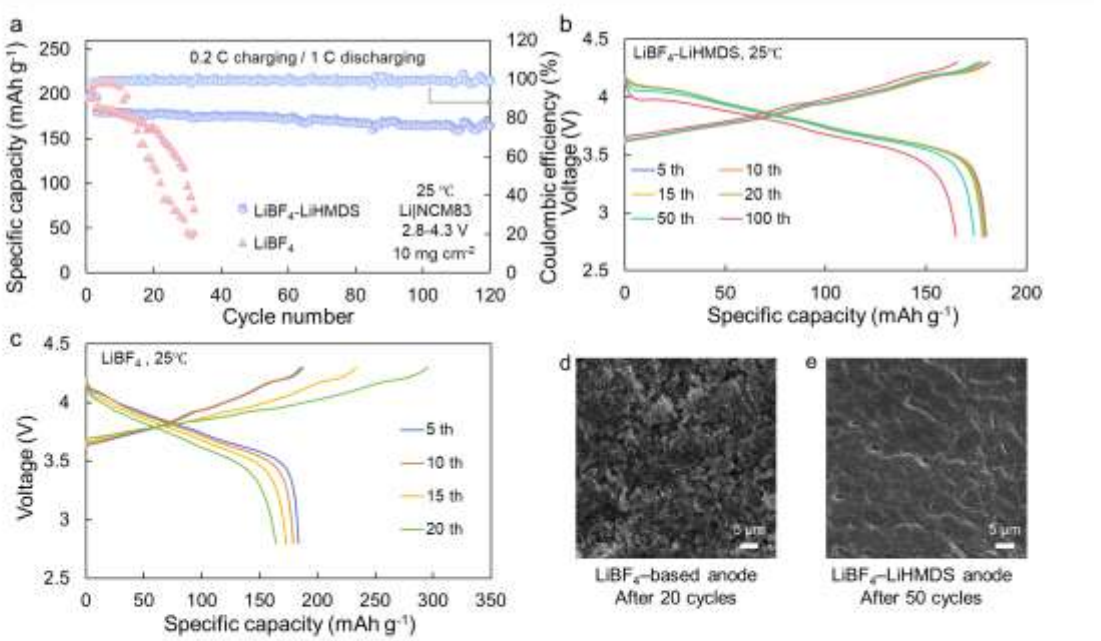

Figure S43. a, b, c cycling performance (a) and the voltage profiles (b-c) of Li||NMC83 using $LiBF_4$ and $LiBF_4$-LiHMDS at room temperature with a charge/discharge rate of 0.2/1 C. SEM images of cycled Li metal in (d) $LiBF_4$ and (e) $LiBF_4$-LiHMDS electrolyte at room temperature.

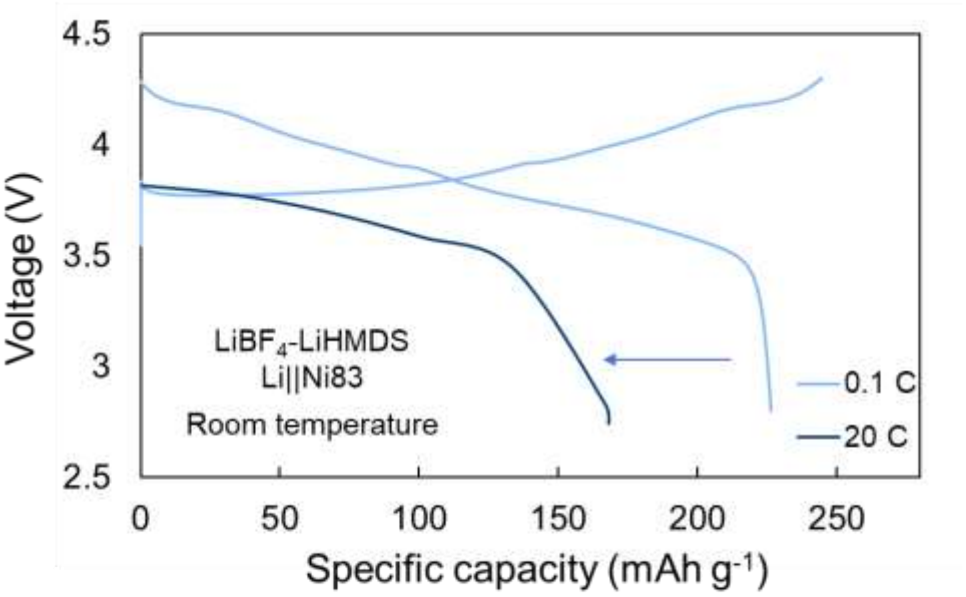

Figure S44. The voltage profiles of the Li||NCM83 batteries at 20 C rate with $LiBF_4$-LiHMDS electrolytes under room temperature.

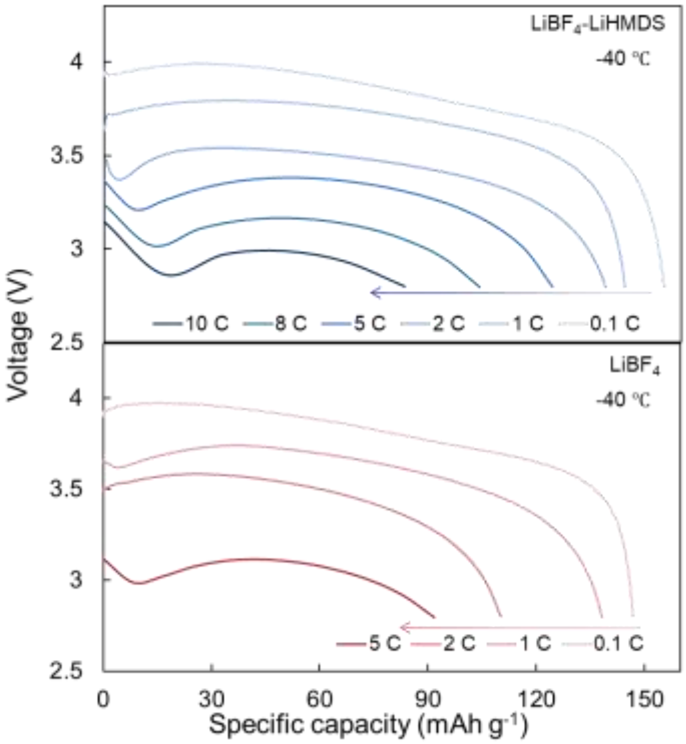

Figure S45. The voltage profiles of the Li||NCM83 batteries at different rates with $LiBF_4$-LiHMDS and $LiBF_4$ electrolytes under −40℃.

Note: During battery discharge at low temperatures or high rates, the voltage initially decreases due to concentration polarization caused by restricted ionic transport and sluggish interfacial kinetics. Subsequently, the exothermic nature of the discharge process reduces polarization, resulting in a concave peak on the discharge curve [16].

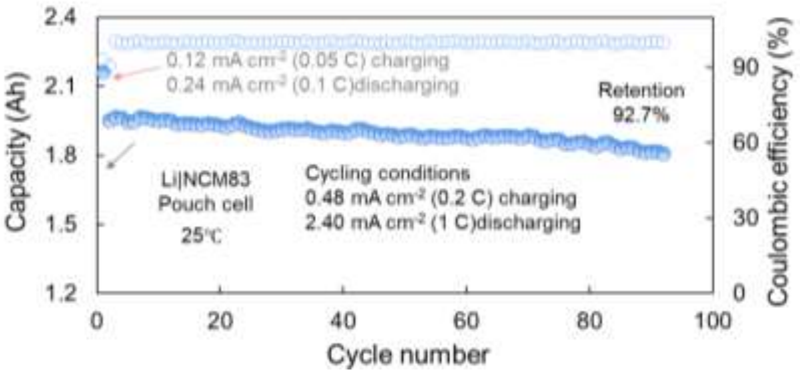

Figure S46. Cycling performance of 2.1 Ah Li||NMC83 pouch cells charged at 0.48 mA cm^{-2} and discharged at 2.40 mA cm^{-2} at 25 ℃. (areal capacity: 2.40 mAh cm^{-2}).

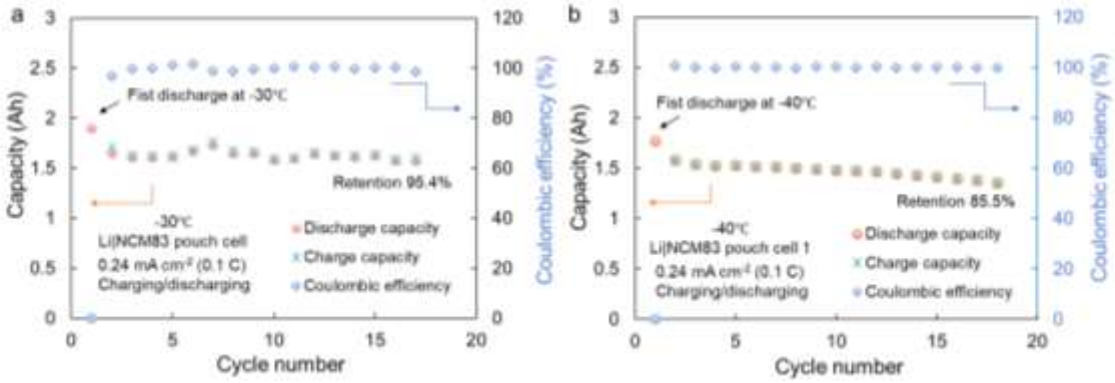

Figure S47. Low-temperature cycling performance of 2.1 Ah Li||NMC83 pouch cells charged at 0.24 mA cm^{-2} and discharged at 0.24 mA cm^{-2} at (a) –30 ℃ and (b) –40 ℃. (areal capacity: 2.40 mAh cm^{-2}).

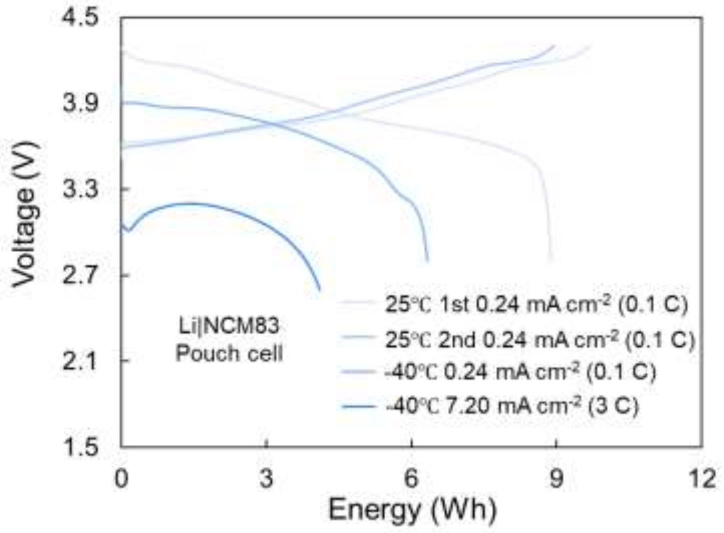

Figure S48. Voltage-energy curves of 2.1 Ah Li|NMC83 pouch cells with $LiBF_4$ -LiHMDS electrolyte under different conditions (areal capacity: 2.40 mAh cm^{-2}, lean electrolyte 1.6 g Ah^{-1}).

Table S1. The calculation details of polarization voltage with different electrolytes at 25℃ and –40℃.

Electrolyte	The medium voltage (V) @25℃ 1st charge	The medium voltage (V) @25℃ 1st discharge	Polarization voltage (V)
LiTFSI	3.8430 (0.1 C)	3.8356 (0.1 C)	0.0074
$LiBF_4$	3.8351 (0.1 C)	3.8295 (0.1 C)	0.0056
Electrolyte	The medium voltage (V) @25℃ 2nd charge	The medium voltage (V) @–40℃ discharge	Polarization voltage (V)
LiTFSI	3.8687 (0.1 C)	2.8000 (0.1 C)	1.0687
$LiBF_4$	3.8550 (0.1 C)	3.6846 (0.1 C)	0.1704
$LiBF_4$	3.7926 (0.1 C)	2.8874 (0.5 C)	0.9052

Table S2. Calculated consumption of electrolyte additives at each electrode based on elemental quantification derived from ICP-OES.

Sample	The mass of the sample portion taken m_0 (g)	Constant volume V_0 (mL)	Original element concentration C_1 (mg/L)	Elemental mass fraction W (%)	Si elemental content of sample (mg)	LiHMDS consumption m_c (mg)
Li anode	0.0150	25	0.377	0.0629%	0.0333	0.0993
NCM83 cathode	0.00500	25	0.930	0.465%	0.0698	0.208

Table S3. The discharge capacity retention under specified conditions and cycling performance comparison of lithium batteries at subzero conditions using different electrolytes. (Note: the discharge capacity retention is defined as the ratio of the actual discharge capacity under specified low-temperature conditions to the rated capacity at room temperature)

Electrolytes	Temperatures	Rate	Discharge capacity retention	Cycling performance	Reference
LiFSI/EmimFSI/dFBn	–20℃	0.2 C	79.5%	100	[22]
$LiBF_4$/DOL/DME/Si-NCO	–40℃	0.1 C	80.0%	150	[23]
	–40℃	1 C	38.6%	/	
LiFSI/TFMP/DME	–40℃	0.5 C	30.0%	100	[24]
LiDFOB/DME/$LiNO_3$/VC	–20℃	0.5 C	81.1%	250	[25]
LiFSI/BFE	–30℃	0.1 C	74.1%	150	[26]
LiFSI/2-MeTHF/TTE	–30℃	0.1 C	52.9%	250	[27]
LiFSI/BTFE/DME	–40℃	0.2 C	54.5%	200	[28]
$LiPF_6$/EC/DMC/$LiNO_3$/HFT	–20℃	0.5 C	66.7%	300	[29]
LiFSI/DMM	–40℃	0.1 C	57.1%	100	[30]
$LiBF_4$/DOL/DME/LiHMDS	–40℃	0.1 C	81.7%	410	This work
		1 C	76.0%	/	
		2 C	73.2%	/	
		5 C	65.4%	/	
		8 C	59.8%	/	
		10 C	43.9%	/	

Table S4. Comparison of our work with recent works on low-temperature lithium-based pouch cells. (Note: the discharge capacity retention is defined as the ratio of the actual discharge capacity under specified low-temperature conditions to the rated capacity at room temperature)

Electrolytes	Cell conditions	Temperatures	Energy density	Power density	Discharge capacity retention (Calculated)	Reference
1M LiFSI in BTFE/DME (5:1)	5Ah Li/NCM811 pouch cell 2.0 mAh cm^{-2}	–20℃	250 Wh kg^{-1}	50 W kg^{-1} (Calculated)	80.5%	[28]
		-40℃	230 Wh kg^{-1}	46 W kg^{-1} (Calculated)	76.5%	
1M LiFSI in DiFEC/MTFC/HFME (1:2:2 vol%)	2Ah Graphite/NCM523 pouch cell 4.0 mAh cm^{-2}	-40℃	270 Wh kg^{-1}	13.5 W kg^{-1} (Calculated)	46.5%	[31]
1M LiFSI in DEE	160 mAh Li/SPAN pouch cell 6.0 mAh cm^{-2}	-40℃	143 Wh kg^{-1}	12 W kg^{-1}	75.0%	[32]
0.6M LiFSI+0.4M LiDFOB in DMS	1Ah Graphite/LCO pouch cell 2.0 mAh cm^{-2}	-20℃	194 Wh kg^{-1}	97 W kg^{-1} (Calculated)	94.0%	[33]
$LiPF_6$ in PC/TFEP/EMC	4Ah Graphite/NCM811 pouch cell 7.2 mAh cm^{-2}	-40℃	221 Wh kg^{-1} (Calculated)	22.1 W kg^{-1} (Calculated)	78.7%	[34]
1M LiFSI-$LiNO_3$ in THP/FEC (95:5 vol%)	2.7Ah Li/NCM811 pouch cell 2.0 mAh cm^{-2}	-20℃	320 Wh kg^{-1}	32 W kg^{-1}	85.2%	[35]
		-40℃	200 Wh kg^{-1}	20 W kg^{-1}	64.8%	
1M LiFSI+0.2 M $LiNO_3$ +0.3M LiSTFSI in DME	3.5Ah Li/NCM811 pouch cell 4.0 mAh cm^{-2}	-40℃	321.1 Wh kg^{-1}	43.1 W kg^{-1}	76.2%	[36]
		-40℃	206.1 Wh kg^{-1}	721.7 W kg^{-1}	57.1%	
2M $LiBF_4$+LiHMDS in DME/DOL	2.1Ah Li/NCM83 pouch cell 2.4 mAh cm^{-2}	-40℃	310.4 Wh kg^{-1}	38.5 W kg^{-1}	82.4%	This work
		-40℃	208.3 Wh kg^{-1}	980.9 W kg^{-1}	66.7%	

REFERENCES

1. Adams BD, Zheng J, Ren X *et al.* Accurate determination of coulombic efficiency for lithium metal anodes and lithium metal batteries. *Adv Energy Mater* 2017; **8**: 1702097.
2. Ge M, Coburn DS, Nazaretski E *et al.* One-minute nano-tomography using hard X-ray full-field transmission microscope. *Appl Phys Lett* 2018; **113**: 083109.
3. Lu X, Bertei A, Finegan DP *et al.* 3D microstructure design of lithium-ion battery electrodes assisted by X-ray nano-computed tomography and modelling. *Nat Commun* 2020; **11**: 2079.
4. Sambasivarao SV, Acevedo O. Development of OPLS-AA force field parameters for 68 unique ionic liquids. *J Chem Theor Comput* 2009; **5**: 1038–50.
5. Becke AD. Density‐functional thermochemistry. III. The role of exact exchange. *J Chem Phys* 1993; **98**: 5648–52.
6. Grimme S, Antony J, Ehrlich S *et al.* A consistent and accurate ab initio parametrization of density functional dispersion correction (DFT-D) for the 94 elements H-Pu. *J Chem Phys* 2010; **132**: 154104.
7. Ding JF, Xu R, Yao N *et al.* Non-solvating and low-dielectricity cosolvent for anion-derived solid electrolyte interphases in lithium metal batteries. *Angew Chem Int Ed* 2021; **60**: 11442-7.
8. Wang D, Lv D, Liu H *et al.* In situ formation of nitrogen-rich solid electrolyte Interphase and simultaneous regulating solvation structures for advanced Zn metal batteries. *Angew Chem Int Ed* 2022; **61**: e202212839.
9. Chen X, Li L, Liu M *et al.* Detection of lithium plating in lithium-ion batteries by distribution of relaxation times. *J Power Sources* 2021; **496**: 229867.
10. Lu Y, Zhao C-Z, Huang J-Q *et al.* The timescale identification decoupling complicated kinetic processes in lithium batteries. *Joule* 2022; **6**: 1172–98.
11. Shafiei Sabet P, Sauer DU. Separation of predominant processes in electrochemical impedance spectra of lithium-ion batteries with nickel-manganese-cobalt cathodes. *J Power Sources* 2019; **425**: 121–9.
12. Liang P, Li J, Dong Y *et al.* Modulating interfacial solvation via ion dipole interactions for low‐temperature and high‐voltage lithium batteries. *Angew Chem Int Ed* 2024; **64**: 202415853.
13. Huang R, Wei G, Wang X *et al.* Revealing the low-temperature aging mechanisms of the whole life cycle for lithium-ion batteries (nickel-cobalt-aluminum vs. graphite). *J Energy Chem* 2025; **106**: 31–43.
14. Rashid M, Gupta A. Effect of relaxation periods over cycling performance of a Li-ion battery. *J Electrochem Soc* 2015; **162**: A3145–A53.
15. Nian Q, Sun T, Liu S *et al.* Issues and opportunities on low-temperature aqueous batteries. *Chem Eng J* 2021; **423**: 130253.
16. Piao N, Gao X, Yang H *et al.* Challenges and development of lithium-ion batteries for low temperature environments. *eTransportation* 2022; **11**: 100145.
17. Xu K, von Cresce A, Lee U. Differentiating contributions to “ion transfer” barrier from interphasial resistance and Li^+ desolvation at electrolyte/graphite interface.

Langmuir 2010; **26**: 11538–43.
18. Yao YX, Chen X, Yao N *et al.* Unlocking charge transfer limitations for extreme fast charging of Li‐ion batteries. *Angew Chem Int Ed* 2022; **62**: 202214828.
19. Liu J, Bao Z, Cui Y *et al.* Pathways for practical high-energy long-cycling lithium metal batteries. *Nat Energy* 2019; **4**: 180–6.
20. Wu M, Wang Z, Zhang W *et al.* High‐performance lithium metal batteries enabled by a fluorinated cyclic ether with a low reduction potential. *Angew Chem Int Ed* 2023; **62**: 202216169.
21. Jia P, Wang J, Zheng T *et al.* Boosting cathode activity and anode stability of lithium–sulfur batteries with vigorous iodic species triggered by nitrate. *Angew Chem Int Ed* 2024; **63**: 202401055.
22. Liu X, Mariani A, Diemant T *et al.* Locally concentrated ionic liquid electrolytes enabling low‐temperature lithium metal batteries. *Angew Chem Int Ed* 2023; **62**: e202305840.
23. Jiang HZ, Yang C, Chen M *et al.* Electrophilically trapping water for preventing polymerization of cyclic ether towards low‐temperature Li metal battery. *Angew Chem Int Ed* 2023; **62**: e202300238.
24. Shi J, Xu C, Lai J *et al.* An amphiphilic molecule‐regulated core‐shell‐solvation electrolyte for Li‐metal batteries at ultra‐low temperature. *Angew Chem Int Ed* 2023; **62**: e202218151.
25. Jiang Z, Yang T, Li C *et al.* Synergistic additives enabling stable cycling of ether electrolyte in 4.4 V Ni‐rich/Li metal batteries. *Adv Funct Mater* 2023; **33**: 2306868.
26. Zhang G, Chang J, Wang L *et al.* A monofluoride ether-based electrolyte solution for fast-charging and low-temperature non-aqueous lithium metal batteries. *Nat Commun* 2023; **14**: 1081.
27. Li X, Li M, Liu Y *et al.* Fast interfacial defluorination kinetics enables stable cycling of low-temperature lithium metal batteries. *J Am Chem Soc* 2024; **146**: 17023–31.
28. Holoubek J, Kim K, Yin Y *et al.* Electrolyte design implications of ion-pairing in low-temperature Li metal batteries. *Energy Environ Sci* 2022; **15**: 1647–58.
29. Jiang J, Li M, Liu X *et al.* Multifunctional additives to realize dendrite‐free lithium deposition in carbonate electrolytes toward low‐temperature Li metal batteries. *Adv Energy Mater* 2024; **14**: 2400365.
30. Ma T, Ni Y, Wang Q *et al.* Optimize lithium deposition at low temperature by weakly solvating power solvent. *Angew Chem Int Ed* 2022; **61**: e202207927.
31. Zheng X, Cao Z, Luo W *et al.* Solvation and interfacial engineering enable −40°C operation of graphite/NCM batteries at energy density over 270 Wh kg^{-1}. *Adv Mater* 2023; **35**: 2210115.
32. Holoubek J, Liu H, Wu Z *et al.* Tailoring electrolyte solvation for Li metal batteries cycled at ultra-low temperature. *Nat Energy* 2021; **6**: 303–13.
33. Zhao Y, Hu Z, Zhao Z *et al.* Strong solvent and dual lithium salts enable fast-charging lithium-ion batteries operating from −78 to 60 °C. *J Am Chem Soc* 2023; **145**: 22184–93.
34. Chen L, Wang J, Chen M *et al.* “Dragging effect” induced fast desolvation

kinetics and −50℃ workable high-safe lithium batteries. *Energy Storage Mater* 2024; **65**: 103098.
35. Li Z, Liao Y, Ji H *et al.* A tetrahydropyran‐based weakly solvating electrolyte for low‐temperature and high‐voltage lithium metal batteries. *Adv Energy Mater* 2025; **15**: 2404120.
36. Lu Y, Cao Q, Zhang W *et al.* Breaking the molecular symmetricity of sulfonimide anions for high-performance lithium metal batteries under extreme cycling conditions. *Nat Energy* 2024; **10**: 191–204.
